# Supplementary figures and images for: Retrospective analysis of spatiotemporal variation of scrub typhus in Yunnan Province, 2006–2022
Source: PLoS Negl Trop Dis. 2024 Dec 10;18(12):e0012654. doi: 10.1371/journal.pntd.0012654 (PMC11630589; doi:10.1371/journal.pntd.0012654)

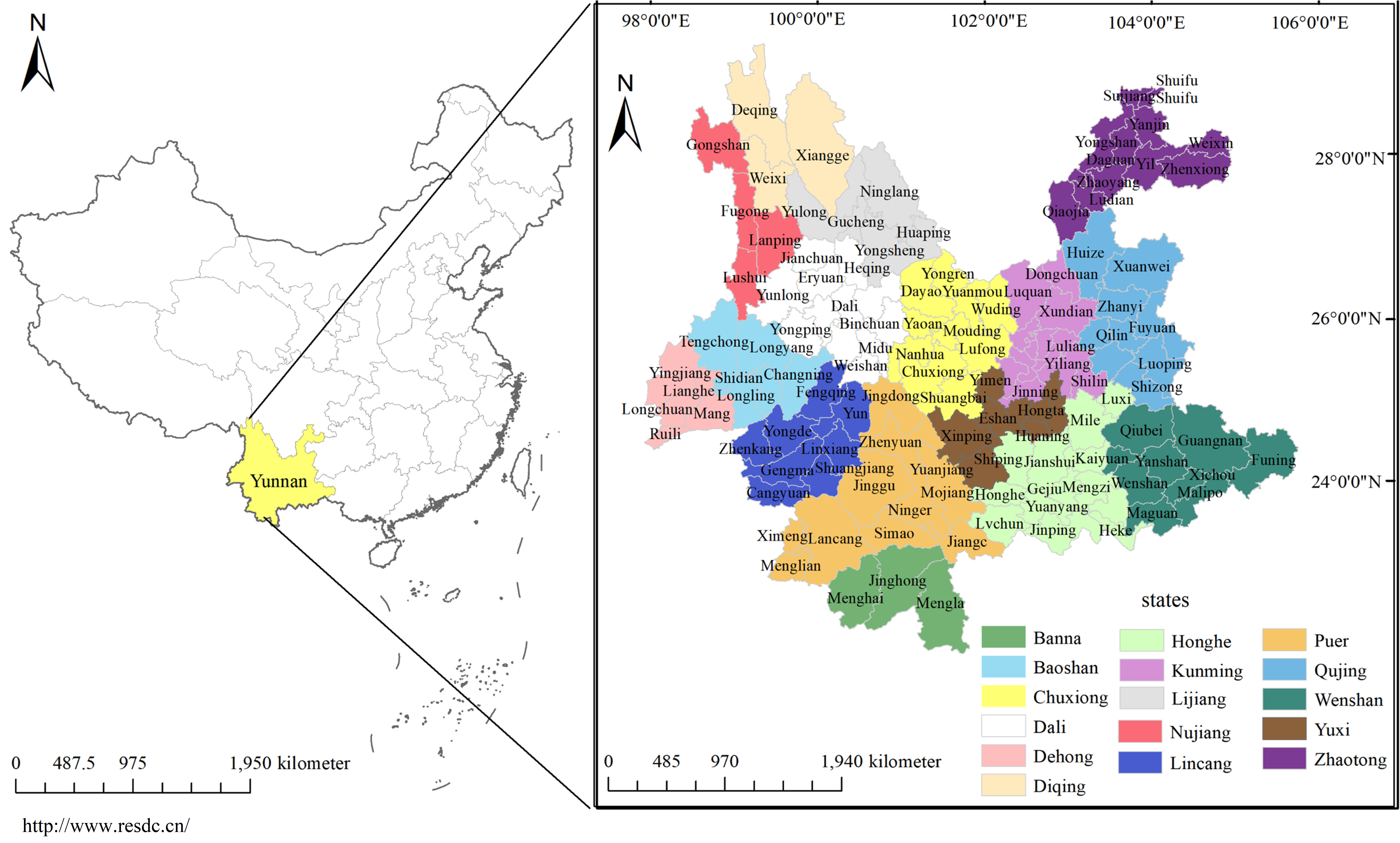

Supplement: S1 Fig — (The county-level map of Yunnan Province used in our study was obtained from the Chinese Academy of Sciences, Institute of Geographic Sciences and Natural Resources Research(http://www.resdc.cn/). This platform allows you to download the required base maps free of charge by registering as a user). (TIF) [file pntd.0012654.s001.tif]

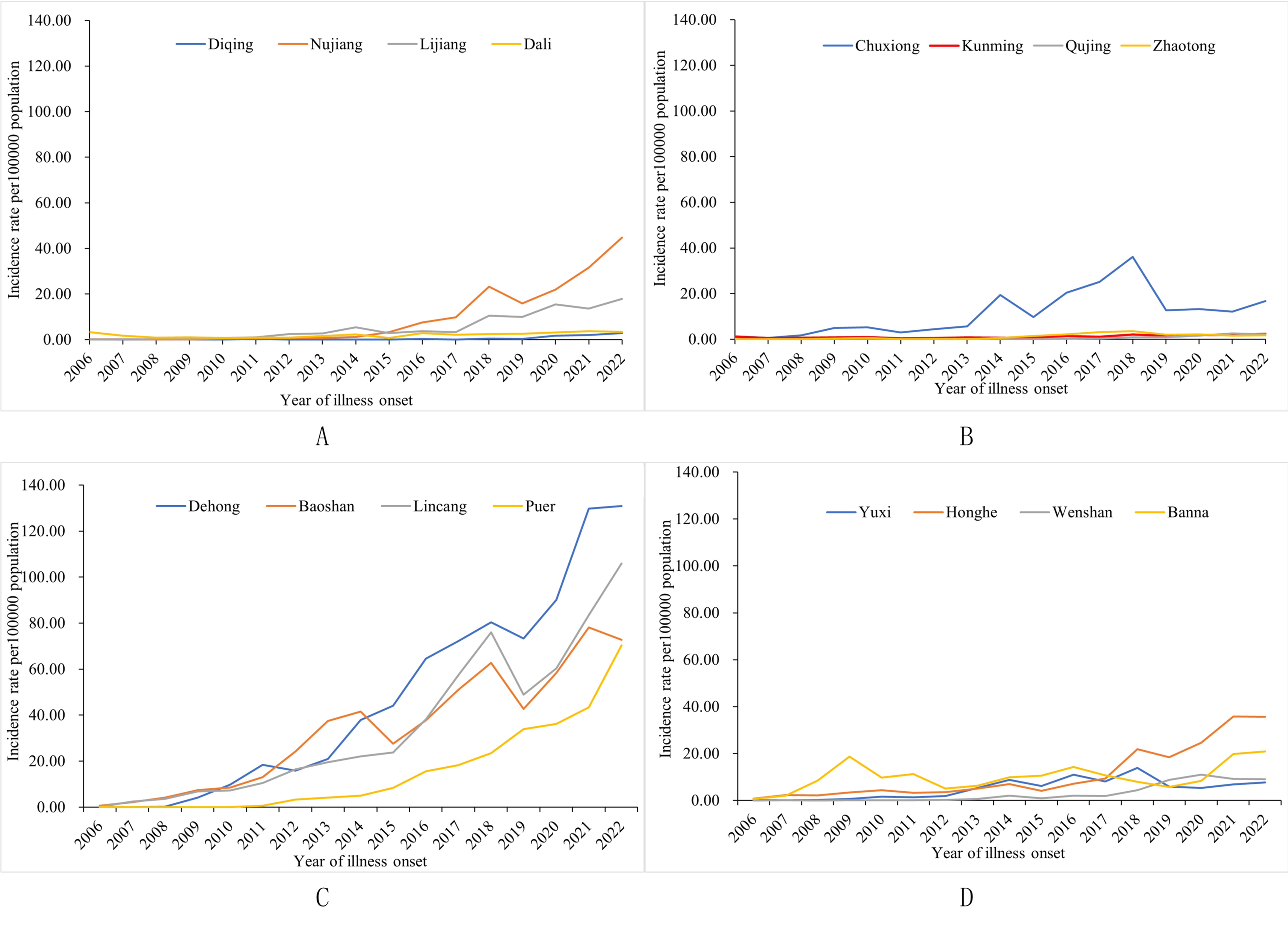

Supplement: S2 Fig — (TIF) [file pntd.0012654.s002.tif]

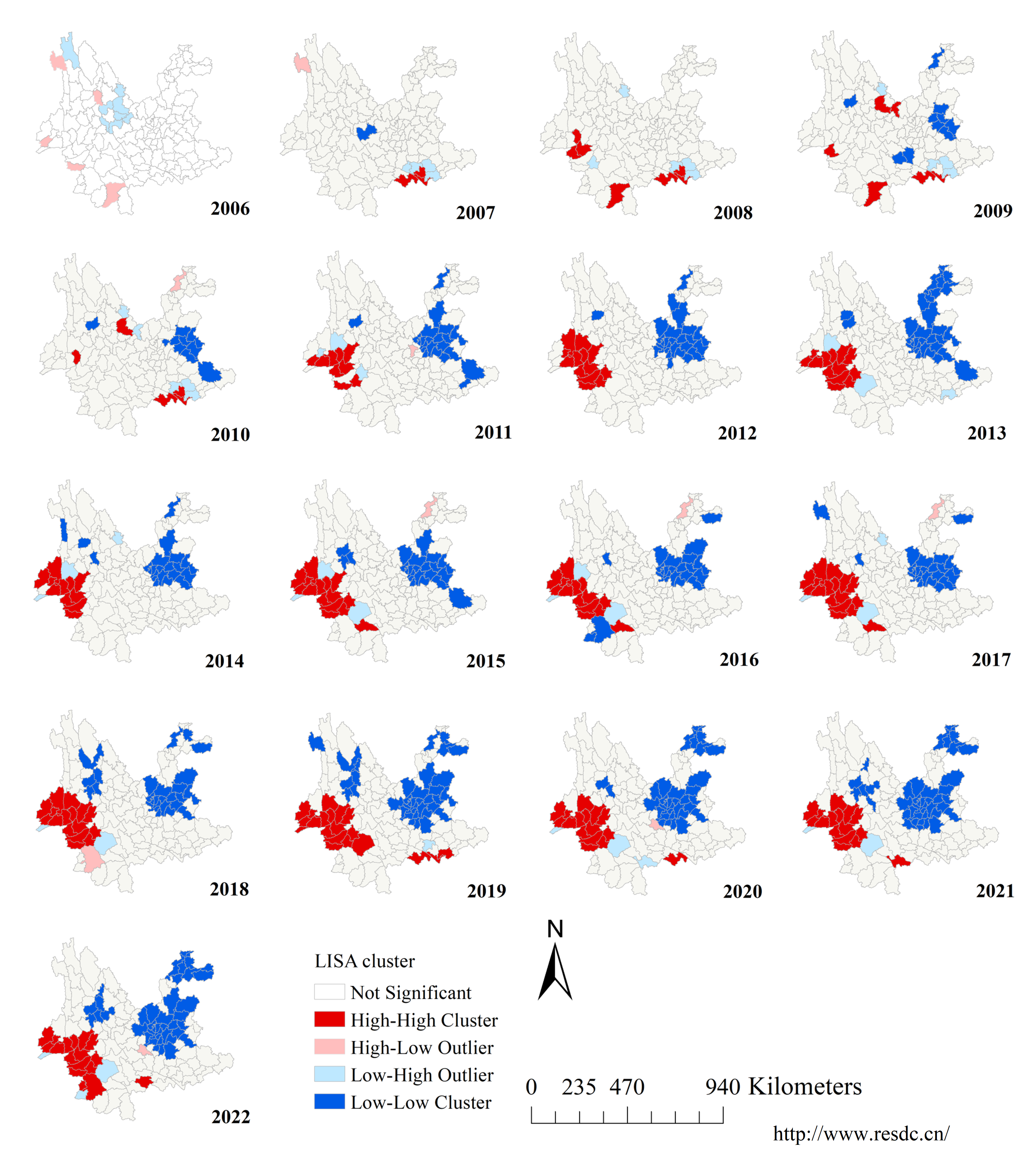

Supplement: S3 Fig — (The county-level map of Yunnan Province used in our study was obtained from the Chinese Academy of Sciences, Institute of Geographic Sciences and Natural Resources Research(http://www.resdc.cn/). This platform allows you to download the required base maps free of charge by registering as a user). (TIF) [file pntd.0012654.s003.tif]
