# Supplementary material for: Retrospective analysis of spatiotemporal variation of scrub typhus in Yunnan Province, 2006–2022
Source: PLoS Negl Trop Dis. 2024 Dec 10;18(12):e0012654. doi: 10.1371/journal.pntd.0012654 (PMC11630589; doi:10.1371/journal.pntd.0012654)
Supplement: S1 Text — (DOCX) [file pntd.0012654.s004.docx]

**S1 Text. Diagnostic criteria for scrub typhus**

**1.Case Diagnosis**

Diagnosis is made based on epidemiological history, clinical manifestations, and laboratory results. In areas endemic to scrub typhus and during the peak season, individuals presenting with unexplained fever or lymph node enlargement should be considered as potential cases of scrub typhus.

**1.1 Epidemiological History**

During the peak season, individuals who have been in or traveled to scrub typhus endemic areas within the preceding 3 weeks and have a history of outdoor activities, mainly involving fieldwork, rural fishing, camping, lying or sitting on grass, and contact with straw, should be considered for scrub typhus.

**1.2 Clinical Manifestations**

1.2.1 Fever

1.2.2 Lymph node enlargement

1.2.3 Rash

1.2.4 Specific eschar or ulcer

**1.3 Laboratory Investigations**

1.3.1 Positive Weil-Felix test: Single serum with OXK titer ≥1:160;

1.3.2 Positive Indirect Immunofluorescence Assay: Four-fold or higher rise in IgG antibody titer in paired sera;

1.3.3 Positive PCR (Polymerase Chain Reaction) for scrub typhus DNA;

1.3.4 Isolation of the causative pathogen.

**2.Diagnostic Criteria**

**2.1 Suspected Cases**

Individuals meeting criteria 1.1 and 1.2.1, plus either 1.2.2 or 1.2.3, with the exclusion of other diseases; or individuals without a clear epidemiological history but presenting with all three criteria 1.2.1, 1.2.2, and 1.2.3 during the peak season.

**2.2 Clinical Diagnosis Cases**

Suspected cases plus criterion 1.2.4; or individuals meeting all three criteria 1.1, 1.2.1, and 1.2.4.

**2.3 Laboratory Diagnosis Cases**

Suspected cases plus any of the criteria 1.3.2, 1.3.3, or 1.3.4; or clinical diagnosis cases plus any of the criteria 1.3.1, 1.3.2, 1.3.3, or 1.3.4.
