# Supplementary material for: Retrospective analysis of spatiotemporal variation of scrub typhus in Yunnan Province, 2006–2022
Source: PLoS Negl Trop Dis. 2024 Dec 10;18(12):e0012654. doi: 10.1371/journal.pntd.0012654 (PMC11630589; doi:10.1371/journal.pntd.0012654)
Supplement: S1 Table — (DOCX) [file pntd.0012654.s005.docx]

**S1 Table. Space-time scan statistical analysis of scrub typhus in Yunnan Province, 2006-2022.**

| **Cluster** | **Coordinates** | **Radius(km)** | **Number of regions** | **Time frame** | **Number of cases** | **Expected cases** | **Relative risk** | **Log-likelihood ratios** | ***P-*value** |
| --- | --- | --- | --- | --- | --- | --- | --- | --- | --- |
| Most likely cluster | (23.762836 N, 98.829611 E) | 144.00 | 7 in Lincang  5 in Dehong  3 in Baoshan | 2014/7/1- 2022/11/30 | 28329 | 3193.40 | 14.09 | 42067.63 | ＜0.001 |
| Secondary cluster 1 | (21.462756 N, 101.558701 E) | 241.56 | 3 in Banna  7 in Puer  4 in Honghe | 2018/6/1- 2022/11/30 | 9300 | 1689.91 | 6.18 | 8683.10 | ＜0.001 |
| Secondary cluster 2 | (25.202931 N, 101.278476 E) | 67.28 | 6 in Chuxiong | 2014/8/1- 2022/10/31 | 3665 | 1213.63 | 3.13 | 1642.75 | ＜0.001 |
| Secondary cluster 3 | (23.132791 N, 104.697673 E) | 105.39 | 5 in Wenshan  2 in Honghe | 2018/5/1- 2022/11/30 | 2571 | 874.73 | 3.01 | 1096.27 | ＜0.001 |
| Secondary cluster 4 | (24.112853 N, 102.728557 E) | 84.86 | 8 in Yuxi  4 in Honghe  1 in Kunming | 2018/7/1- 2018/9/30 | 410 | 95.55 | 4.31 | 283.40 | ＜0.001 |
| Secondary cluster 5 | (26.113473 N, 99.959014 E) | 65.76 | 6 in Dali  1 in Nujiang | 2021/8/1- 2021/10/31 | 119 | 47.40 | 2.51 | 37.97 | ＜0.001 |
