# Supplementary material for: Retrospective analysis of spatiotemporal variation of scrub typhus in Yunnan Province, 2006–2022
Source: PLoS Negl Trop Dis. 2024 Dec 10;18(12):e0012654. doi: 10.1371/journal.pntd.0012654 (PMC11630589; doi:10.1371/journal.pntd.0012654)
Supplement: S1 Data — (DOCX) [file pntd.0012654.s006.docx]

| Tabel 1.Reported scrub typhus cases, Yunnan, 2006–2022, yearly | | | | | | | | | | | | | | | | | | |
| --- | --- | --- | --- | --- | --- | --- | --- | --- | --- | --- | --- | --- | --- | --- | --- | --- | --- | --- |
| Prefecture | 2006 | 2007 | 2008 | 2009 | 2010 | 2011 | 2012 | 2013 | 2014 | 2015 | 2016 | 2017 | 2018 | 2019 | 2020 | 2021 | 2022 | Totle |
| Kunming | 68 | 29 | 37 | 51 | 58 | 28 | 35 | 52 | 48 | 56 | 92 | 76 | 144 | 100 | 138 | 168 | 208 | 1388 |
| Qujing | 2 | 2 | 1 | 0 | 1 | 3 | 2 | 9 | 4 | 4 | 12 | 21 | 56 | 58 | 95 | 148 | 124 | 542 |
| Yuxi | 6 | 3 | 4 | 14 | 35 | 30 | 42 | 123 | 206 | 146 | 260 | 193 | 330 | 140 | 124 | 152 | 174 | 1982 |
| Baoshan | 14 | 53 | 103 | 181 | 211 | 327 | 614 | 953 | 1064 | 708 | 978 | 1327 | 1642 | 1121 | 1476 | 1892 | 1744 | 14408 |
| Zhaotong | 1 | 0 | 0 | 15 | 33 | 4 | 9 | 3 | 29 | 78 | 119 | 169 | 200 | 115 | 111 | 80 | 84 | 1050 |
| Lijiang | 1 | 1 | 1 | 2 | 8 | 12 | 30 | 35 | 68 | 36 | 47 | 42 | 135 | 129 | 198 | 171 | 221 | 1137 |
| Puer | 0 | 0 | 1 | 2 | 2 | 14 | 85 | 106 | 130 | 217 | 406 | 479 | 619 | 896 | 914 | 1038 | 1667 | 6576 |
| Lincang | 5 | 57 | 83 | 162 | 176 | 255 | 403 | 483 | 548 | 595 | 960 | 1447 | 1923 | 1241 | 1445 | 1875 | 2371 | 14029 |
| Chuxiong | 35 | 17 | 48 | 132 | 140 | 82 | 120 | 154 | 527 | 264 | 558 | 691 | 992 | 347 | 343 | 290 | 397 | 5137 |
| Honghe | 36 | 101 | 97 | 152 | 197 | 149 | 162 | 231 | 318 | 188 | 333 | 439 | 1033 | 879 | 1136 | 1592 | 1567 | 8610 |
| Wenshan | 0 | 1 | 0 | 3 | 5 | 1 | 8 | 20 | 74 | 33 | 74 | 66 | 157 | 322 | 392 | 318 | 311 | 1785 |
| Banna | 6 | 21 | 91 | 201 | 107 | 128 | 58 | 73 | 114 | 123 | 167 | 126 | 94 | 69 | 105 | 258 | 273 | 2014 |
| Dali | 113 | 60 | 31 | 37 | 25 | 29 | 32 | 57 | 79 | 26 | 101 | 76 | 89 | 91 | 111 | 121 | 111 | 1189 |
| Dehong | 2 | 1 | 2 | 49 | 117 | 224 | 194 | 259 | 475 | 560 | 830 | 939 | 1054 | 967 | 1190 | 1709 | 1729 | 10301 |
| Nujiang | 1 | 1 | 0 | 0 | 2 | 2 | 4 | 4 | 6 | 18 | 41 | 53 | 128 | 88 | 122 | 175 | 244 | 889 |
| Diqing | 0 | 0 | 0 | 0 | 0 | 1 | 0 | 0 | 0 | 0 | 1 | 0 | 2 | 1 | 7 | 8 | 11 | 31 |
| Total | 290 | 347 | 499 | 1001 | 1117 | 1289 | 1798 | 2562 | 3690 | 3052 | 4979 | 6144 | 8598 | 6564 | 7907 | 9995 | 11236 | 71068 |

| Tabel 2.Reported scrub typhus cases, Yunnan, 2006–2022, monthly | | | | | | | | | | | | | |
| --- | --- | --- | --- | --- | --- | --- | --- | --- | --- | --- | --- | --- | --- |
| Prefecture | Jan | Feb | Mar | Apr | May | June | July | Aug | Sep | Oct | Nov | Dec | Total |
| Kunming | 3 | 6 | 16 | 12 | 18 | 44 | 304 | 454 | 318 | 149 | 46 | 18 | 1388 |
| Qujing | 2 | 6 | 4 | 7 | 12 | 56 | 134 | 114 | 80 | 72 | 42 | 13 | 542 |
| Yuxi | 8 | 4 | 9 | 12 | 22 | 69 | 382 | 635 | 493 | 259 | 65 | 24 | 1982 |
| Baoshan | 61 | 21 | 42 | 50 | 112 | 730 | 2753 | 4532 | 3247 | 2124 | 579 | 157 | 14408 |
| Zhaotong | 1 | 1 | 4 | 4 | 15 | 73 | 228 | 350 | 224 | 133 | 11 | 6 | 1050 |
| Lijiang | 6 | 3 | 4 | 3 | 10 | 30 | 172 | 401 | 294 | 164 | 36 | 14 | 1137 |
| Puer | 125 | 74 | 43 | 57 | 246 | 625 | 1148 | 1121 | 940 | 1092 | 772 | 333 | 6576 |
| Lincang | 92 | 27 | 35 | 62 | 186 | 956 | 2545 | 3313 | 2842 | 2386 | 1232 | 353 | 14029 |
| Chuxiong | 63 | 26 | 18 | 26 | 37 | 111 | 614 | 1503 | 1549 | 875 | 228 | 87 | 5137 |
| Honghe | 35 | 23 | 38 | 103 | 420 | 1101 | 1743 | 1988 | 1443 | 922 | 600 | 194 | 8610 |
| Wenshan | 10 | 7 | 8 | 15 | 90 | 259 | 402 | 408 | 249 | 208 | 96 | 33 | 1785 |
| Banna | 60 | 36 | 15 | 32 | 149 | 277 | 370 | 275 | 229 | 263 | 204 | 104 | 2014 |
| Dali | 11 | 2 | 6 | 8 | 9 | 65 | 259 | 338 | 303 | 153 | 26 | 9 | 1189 |
| Dehong | 110 | 45 | 37 | 45 | 174 | 930 | 2128 | 2280 | 1630 | 1703 | 953 | 266 | 10301 |
| Nujiang | 4 | 0 | 0 | 4 | 3 | 23 | 174 | 256 | 219 | 175 | 27 | 4 | 889 |
| Diqing | 0 | 0 | 0 | 0 | 0 | 2 | 8 | 11 | 5 | 2 | 3 | 0 | 31 |
| Total | 591 | 281 | 279 | 440 | 1503 | 5351 | 13364 | 17979 | 14065 | 10680 | 4920 | 1615 | 71068 |

| Tabel 3.Reported scrub typhus cases, Yunnan, 2006–2022 | | | | | | | | | | | | | | | | | | |
| --- | --- | --- | --- | --- | --- | --- | --- | --- | --- | --- | --- | --- | --- | --- | --- | --- | --- | --- |
| Counties | 2006 | 2007 | 2008 | 2009 | 2010 | 2011 | 2012 | 2013 | 2014 | 2015 | 2016 | 2017 | 2018 | 2019 | 2020 | 2021 | 2022 | Total |
| Chenggong | 23 | 7 | 6 | 11 | 3 | 0 | 2 | 4 | 3 | 2 | 7 | 4 | 10 | 7 | 5 | 18 | 29 | 141 |
| Wuhua | 1 | 0 | 0 | 1 | 2 | 1 | 0 | 2 | 1 | 3 | 1 | 1 | 5 | 4 | 2 | 3 | 4 | 31 |
| Panlong | 4 | 0 | 0 | 1 | 1 | 1 | 0 | 0 | 2 | 2 | 6 | 5 | 4 | 7 | 8 | 8 | 7 | 56 |
| Guandu | 7 | 3 | 4 | 5 | 2 | 2 | 6 | 2 | 7 | 13 | 5 | 6 | 6 | 7 | 9 | 6 | 11 | 101 |
| Xishan | 6 | 4 | 2 | 2 | 10 | 2 | 6 | 7 | 1 | 1 | 8 | 8 | 18 | 8 | 11 | 15 | 12 | 121 |
| Jinning | 2 | 0 | 1 | 0 | 0 | 2 | 0 | 1 | 2 | 3 | 7 | 2 | 3 | 7 | 10 | 15 | 25 | 80 |
| Dongchuan | 0 | 3 | 0 | 1 | 2 | 1 | 0 | 1 | 1 | 1 | 1 | 1 | 0 | 1 | 0 | 0 | 2 | 15 |
| Songming | 1 | 1 | 1 | 1 | 3 | 0 | 0 | 0 | 3 | 0 | 3 | 1 | 6 | 2 | 4 | 1 | 4 | 31 |
| Yiliang | 1 | 0 | 0 | 1 | 1 | 1 | 0 | 1 | 2 | 3 | 3 | 4 | 5 | 4 | 4 | 3 | 4 | 37 |
| Fuming | 0 | 0 | 0 | 0 | 0 | 0 | 0 | 0 | 0 | 0 | 0 | 0 | 1 | 1 | 0 | 2 | 3 | 7 |
| Shilin | 0 | 0 | 0 | 0 | 0 | 0 | 0 | 0 | 0 | 1 | 0 | 4 | 2 | 2 | 3 | 4 | 2 | 18 |
| Luquan | 1 | 2 | 5 | 9 | 11 | 5 | 4 | 6 | 6 | 8 | 16 | 13 | 43 | 20 | 29 | 37 | 45 | 260 |
| Xundian | 0 | 0 | 1 | 0 | 0 | 0 | 0 | 0 | 2 | 2 | 1 | 2 | 3 | 0 | 6 | 3 | 5 | 25 |
| Anning | 22 | 9 | 17 | 19 | 23 | 13 | 17 | 28 | 18 | 17 | 34 | 25 | 38 | 30 | 47 | 53 | 55 | 465 |
| Qilin | 0 | 0 | 0 | 0 | 0 | 0 | 0 | 0 | 0 | 0 | 1 | 0 | 1 | 0 | 2 | 3 | 1 | 8 |
| Zhanyi | 1 | 0 | 0 | 0 | 0 | 0 | 0 | 0 | 0 | 0 | 0 | 1 | 0 | 0 | 0 | 0 | 1 | 3 |
| Xuanwei | 0 | 0 | 0 | 0 | 0 | 0 | 0 | 1 | 0 | 0 | 0 | 3 | 5 | 1 | 5 | 4 | 7 | 26 |
| Luoping | 0 | 0 | 0 | 0 | 0 | 0 | 0 | 1 | 0 | 0 | 2 | 6 | 16 | 12 | 28 | 40 | 42 | 147 |
| Fuyuan | 0 | 0 | 0 | 0 | 0 | 0 | 0 | 0 | 1 | 0 | 3 | 0 | 0 | 0 | 4 | 1 | 4 | 13 |
| Shizong | 0 | 1 | 0 | 0 | 0 | 2 | 1 | 6 | 1 | 4 | 4 | 7 | 31 | 41 | 46 | 96 | 63 | 303 |
| Luliang | 1 | 1 | 0 | 0 | 0 | 0 | 0 | 0 | 0 | 0 | 0 | 1 | 0 | 1 | 4 | 1 | 1 | 10 |
| Huize | 0 | 0 | 1 | 0 | 1 | 1 | 1 | 1 | 2 | 0 | 2 | 3 | 3 | 3 | 6 | 2 | 4 | 30 |
| Malong | 0 | 0 | 0 | 0 | 0 | 0 | 0 | 0 | 0 | 0 | 0 | 0 | 0 | 0 | 0 | 1 | 1 | 2 |
| Hongta | 5 | 0 | 3 | 8 | 8 | 10 | 13 | 36 | 37 | 41 | 82 | 44 | 96 | 28 | 40 | 51 | 49 | 551 |
| Jiangchuan | 0 | 0 | 0 | 1 | 3 | 2 | 4 | 5 | 3 | 6 | 12 | 6 | 16 | 7 | 8 | 8 | 8 | 89 |
| Chengjiang | 1 | 0 | 0 | 2 | 1 | 0 | 1 | 4 | 7 | 10 | 11 | 11 | 14 | 23 | 7 | 7 | 7 | 106 |
| Tonghai | 0 | 0 | 0 | 1 | 2 | 5 | 2 | 13 | 17 | 17 | 44 | 25 | 43 | 18 | 5 | 13 | 13 | 218 |
| Huaning | 0 | 3 | 0 | 0 | 8 | 3 | 7 | 9 | 20 | 13 | 27 | 13 | 32 | 14 | 9 | 12 | 15 | 185 |
| Yimen | 0 | 0 | 0 | 2 | 0 | 0 | 0 | 5 | 48 | 12 | 16 | 15 | 32 | 7 | 9 | 12 | 8 | 166 |
| Eshan | 0 | 0 | 1 | 0 | 6 | 4 | 2 | 30 | 51 | 36 | 61 | 66 | 72 | 35 | 36 | 37 | 57 | 494 |
| Xinping | 0 | 0 | 0 | 0 | 4 | 4 | 8 | 7 | 15 | 8 | 6 | 5 | 15 | 5 | 7 | 10 | 9 | 103 |
| Yuanjiang | 0 | 0 | 0 | 0 | 3 | 2 | 5 | 14 | 8 | 3 | 1 | 8 | 10 | 3 | 3 | 2 | 8 | 70 |
| Longyang | 6 | 23 | 26 | 45 | 10 | 29 | 62 | 64 | 89 | 58 | 98 | 182 | 246 | 183 | 236 | 279 | 254 | 1890 |
| Shidian | 0 | 4 | 13 | 24 | 24 | 24 | 41 | 94 | 104 | 67 | 77 | 155 | 160 | 124 | 138 | 118 | 125 | 1292 |
| Tengchong | 0 | 15 | 22 | 37 | 57 | 74 | 96 | 188 | 274 | 168 | 181 | 221 | 247 | 212 | 275 | 393 | 358 | 2818 |
| Longling | 1 | 0 | 36 | 45 | 61 | 171 | 366 | 484 | 489 | 325 | 414 | 480 | 574 | 414 | 556 | 727 | 602 | 5745 |
| Changning | 7 | 11 | 6 | 30 | 59 | 29 | 49 | 123 | 108 | 90 | 208 | 289 | 415 | 188 | 271 | 375 | 405 | 2663 |
| Zhaoyang | 1 | 0 | 0 | 0 | 1 | 0 | 0 | 0 | 2 | 4 | 13 | 21 | 16 | 19 | 11 | 7 | 13 | 108 |
| Shuifu | 0 | 0 | 0 | 0 | 0 | 0 | 0 | 0 | 0 | 0 | 0 | 0 | 0 | 0 | 0 | 0 | 0 | 0 |
| Yongshan | 0 | 0 | 0 | 11 | 27 | 4 | 7 | 2 | 24 | 59 | 67 | 82 | 66 | 42 | 16 | 19 | 18 | 444 |
| Daguan | 0 | 0 | 0 | 0 | 0 | 0 | 0 | 0 | 0 | 0 | 0 | 0 | 1 | 0 | 2 | 2 | 2 | 7 |
| Ludian | 0 | 0 | 0 | 1 | 0 | 0 | 0 | 0 | 1 | 2 | 11 | 2 | 19 | 2 | 3 | 9 | 7 | 57 |
| Yanjin | 0 | 0 | 0 | 0 | 0 | 0 | 1 | 0 | 0 | 0 | 1 | 0 | 2 | 0 | 2 | 0 | 1 | 7 |
| suijiang | 0 | 0 | 0 | 0 | 1 | 0 | 0 | 1 | 0 | 0 | 0 | 0 | 0 | 0 | 0 | 1 | 0 | 3 |
| Yiliang | 0 | 0 | 0 | 0 | 0 | 0 | 0 | 0 | 0 | 2 | 0 | 0 | 0 | 0 | 0 | 0 | 2 | 4 |
| Weixin | 0 | 0 | 0 | 0 | 0 | 0 | 0 | 0 | 0 | 0 | 0 | 0 | 0 | 0 | 0 | 0 | 0 | 0 |
| Qiaojia | 0 | 0 | 0 | 3 | 4 | 0 | 1 | 0 | 2 | 10 | 27 | 64 | 95 | 50 | 67 | 39 | 41 | 403 |
| Zhenxiong | 0 | 0 | 0 | 0 | 0 | 0 | 0 | 0 | 0 | 1 | 0 | 0 | 1 | 2 | 10 | 3 | 0 | 17 |
| Gucheng | 0 | 0 | 0 | 0 | 0 | 0 | 0 | 1 | 3 | 2 | 1 | 1 | 0 | 1 | 1 | 0 | 1 | 11 |
| Yulong | 0 | 0 | 0 | 0 | 0 | 0 | 0 | 4 | 8 | 20 | 1 | 0 | 3 | 0 | 0 | 1 | 3 | 40 |
| Yongsheng | 0 | 1 | 1 | 2 | 7 | 12 | 30 | 25 | 49 | 8 | 21 | 33 | 32 | 23 | 30 | 24 | 23 | 321 |
| Huaping | 1 | 0 | 0 | 0 | 1 | 0 | 0 | 4 | 8 | 6 | 23 | 8 | 95 | 102 | 165 | 143 | 185 | 741 |
| Ninglang | 0 | 0 | 0 | 0 | 0 | 0 | 0 | 1 | 0 | 0 | 1 | 0 | 5 | 3 | 2 | 3 | 9 | 24 |
| Simao | 0 | 0 | 0 | 0 | 0 | 4 | 1 | 1 | 29 | 45 | 81 | 174 | 167 | 172 | 165 | 197 | 117 | 1153 |
| Ninger | 0 | 0 | 0 | 0 | 0 | 2 | 1 | 54 | 58 | 141 | 231 | 253 | 272 | 181 | 195 | 144 | 213 | 1745 |
| Mojiang | 0 | 0 | 1 | 0 | 1 | 7 | 42 | 12 | 28 | 12 | 31 | 13 | 14 | 13 | 31 | 17 | 46 | 268 |
| Jingdong | 0 | 0 | 0 | 0 | 0 | 0 | 2 | 2 | 2 | 0 | 7 | 4 | 9 | 11 | 8 | 11 | 13 | 69 |
| Jinggu | 0 | 0 | 0 | 0 | 0 | 0 | 6 | 10 | 5 | 10 | 15 | 12 | 19 | 78 | 33 | 19 | 51 | 258 |
| Zhenyuan | 0 | 0 | 0 | 0 | 1 | 0 | 4 | 16 | 6 | 3 | 38 | 4 | 6 | 12 | 19 | 10 | 10 | 129 |
| Jiangcheng | 0 | 0 | 0 | 0 | 0 | 0 | 1 | 5 | 0 | 3 | 1 | 8 | 5 | 12 | 2 | 81 | 148 | 266 |
| Menglian | 0 | 0 | 0 | 0 | 0 | 0 | 1 | 0 | 1 | 0 | 0 | 0 | 0 | 6 | 2 | 3 | 3 | 16 |
| Lancang | 0 | 0 | 0 | 1 | 0 | 0 | 16 | 6 | 1 | 0 | 2 | 10 | 127 | 411 | 456 | 520 | 855 | 2405 |
| Ximeng | 0 | 0 | 0 | 1 | 0 | 1 | 11 | 0 | 0 | 3 | 0 | 1 | 0 | 0 | 3 | 36 | 211 | 267 |
| Linxiang | 0 | 5 | 2 | 5 | 11 | 10 | 40 | 74 | 75 | 62 | 109 | 123 | 203 | 119 | 150 | 217 | 215 | 1420 |
| Fengqing | 1 | 0 | 0 | 0 | 1 | 3 | 9 | 50 | 53 | 42 | 80 | 160 | 275 | 123 | 126 | 172 | 369 | 1464 |
| Yongde | 0 | 11 | 32 | 51 | 47 | 63 | 98 | 88 | 77 | 77 | 125 | 217 | 269 | 218 | 283 | 279 | 317 | 2252 |
| zhenkang | 0 | 2 | 9 | 12 | 7 | 12 | 17 | 28 | 43 | 57 | 61 | 73 | 162 | 145 | 163 | 173 | 216 | 1180 |
| Yun | 0 | 2 | 0 | 2 | 6 | 5 | 11 | 20 | 37 | 34 | 64 | 122 | 249 | 147 | 157 | 244 | 307 | 1407 |
| Cangyuan | 4 | 9 | 12 | 35 | 13 | 16 | 25 | 41 | 73 | 61 | 132 | 188 | 175 | 127 | 185 | 167 | 195 | 1458 |
| Gengma | 0 | 28 | 28 | 39 | 68 | 114 | 133 | 107 | 113 | 180 | 267 | 352 | 357 | 242 | 207 | 301 | 407 | 2943 |
| shuangjiang | 0 | 0 | 0 | 18 | 23 | 32 | 70 | 75 | 77 | 82 | 122 | 212 | 233 | 120 | 174 | 322 | 345 | 1905 |
| Chuxiong | 11 | 2 | 6 | 10 | 13 | 9 | 27 | 30 | 164 | 71 | 272 | 244 | 458 | 98 | 136 | 91 | 175 | 1817 |
| Shuangbai | 1 | 0 | 0 | 7 | 7 | 3 | 19 | 23 | 55 | 26 | 43 | 70 | 112 | 37 | 30 | 22 | 27 | 482 |
| mouding | 0 | 0 | 0 | 1 | 1 | 0 | 1 | 1 | 2 | 3 | 3 | 3 | 5 | 3 | 0 | 1 | 2 | 26 |
| Nanhua | 0 | 0 | 2 | 1 | 5 | 2 | 5 | 9 | 39 | 12 | 75 | 83 | 160 | 45 | 53 | 38 | 74 | 603 |
| Yaoan | 0 | 0 | 0 | 1 | 3 | 1 | 0 | 0 | 1 | 0 | 2 | 2 | 2 | 1 | 0 | 3 | 2 | 18 |
| Dayao | 2 | 0 | 18 | 35 | 43 | 24 | 19 | 58 | 139 | 68 | 129 | 112 | 144 | 102 | 91 | 99 | 84 | 1167 |
| Yongren | 20 | 14 | 20 | 65 | 57 | 39 | 38 | 25 | 105 | 70 | 24 | 155 | 77 | 30 | 13 | 17 | 21 | 790 |
| Yuanmou | 1 | 1 | 1 | 8 | 4 | 2 | 1 | 0 | 4 | 2 | 1 | 4 | 2 | 3 | 0 | 2 | 2 | 38 |
| Wuding | 0 | 0 | 0 | 3 | 6 | 1 | 5 | 6 | 10 | 8 | 5 | 7 | 16 | 8 | 7 | 4 | 2 | 88 |
| Lufeng | 0 | 0 | 1 | 1 | 1 | 1 | 5 | 2 | 8 | 4 | 4 | 11 | 16 | 20 | 13 | 13 | 8 | 108 |
| Mengzi | 0 | 0 | 3 | 0 | 0 | 2 | 3 | 1 | 5 | 3 | 4 | 17 | 36 | 30 | 16 | 29 | 30 | 179 |
| Gejiu | 0 | 0 | 1 | 0 | 1 | 0 | 1 | 0 | 1 | 0 | 0 | 0 | 1 | 8 | 14 | 24 | 29 | 80 |
| Kaiyuan | 2 | 0 | 9 | 21 | 5 | 0 | 1 | 1 | 5 | 1 | 10 | 12 | 25 | 19 | 13 | 3 | 15 | 142 |
| Mile | 1 | 0 | 16 | 8 | 6 | 3 | 9 | 4 | 23 | 22 | 60 | 55 | 108 | 77 | 64 | 58 | 63 | 577 |
| Jianshui | 0 | 0 | 0 | 1 | 1 | 1 | 3 | 1 | 2 | 3 | 6 | 34 | 58 | 37 | 35 | 28 | 31 | 241 |
| Shiping | 0 | 0 | 0 | 1 | 7 | 14 | 9 | 18 | 20 | 10 | 32 | 25 | 51 | 15 | 16 | 9 | 9 | 236 |
| Lvchun | 0 | 0 | 0 | 0 | 2 | 3 | 1 | 1 | 4 | 0 | 0 | 2 | 50 | 66 | 147 | 183 | 134 | 593 |
| Luxi | 0 | 0 | 0 | 0 | 0 | 0 | 0 | 1 | 1 | 0 | 0 | 0 | 4 | 1 | 1 | 7 | 4 | 19 |
| Yuanyang | 1 | 0 | 1 | 0 | 6 | 2 | 5 | 26 | 69 | 57 | 80 | 110 | 239 | 190 | 304 | 400 | 369 | 1859 |
| Honghe | 0 | 0 | 0 | 0 | 1 | 0 | 1 | 2 | 7 | 4 | 13 | 17 | 102 | 73 | 138 | 236 | 259 | 853 |
| Jinping | 23 | 19 | 19 | 16 | 42 | 64 | 60 | 70 | 17 | 21 | 32 | 40 | 82 | 77 | 140 | 362 | 278 | 1362 |
| Hekou | 2 | 25 | 23 | 48 | 83 | 25 | 38 | 66 | 103 | 40 | 55 | 63 | 100 | 112 | 108 | 84 | 102 | 1077 |
| Pingbian | 7 | 57 | 25 | 57 | 43 | 35 | 31 | 40 | 61 | 27 | 41 | 64 | 177 | 174 | 140 | 169 | 244 | 1392 |
| Wenshan | 0 | 0 | 0 | 0 | 0 | 0 | 1 | 0 | 1 | 3 | 3 | 15 | 26 | 39 | 39 | 46 | 73 | 246 |
| Yanshan | 0 | 0 | 0 | 0 | 0 | 0 | 0 | 0 | 3 | 0 | 5 | 1 | 2 | 4 | 2 | 4 | 2 | 23 |
| Xichou | 0 | 0 | 0 | 0 | 0 | 0 | 2 | 0 | 5 | 0 | 5 | 2 | 4 | 7 | 4 | 3 | 1 | 33 |
| Malipo | 0 | 0 | 0 | 0 | 0 | 0 | 2 | 2 | 7 | 2 | 5 | 15 | 36 | 85 | 94 | 81 | 57 | 386 |
| Maguan | 0 | 1 | 0 | 3 | 5 | 1 | 3 | 14 | 42 | 13 | 25 | 16 | 46 | 116 | 159 | 139 | 111 | 694 |
| Qiubei | 0 | 0 | 0 | 0 | 0 | 0 | 0 | 1 | 7 | 8 | 13 | 5 | 8 | 6 | 6 | 7 | 11 | 72 |
| Guangnan | 0 | 0 | 0 | 0 | 0 | 0 | 0 | 3 | 9 | 7 | 16 | 12 | 35 | 62 | 88 | 34 | 31 | 297 |
| Funing | 0 | 0 | 0 | 0 | 0 | 0 | 0 | 0 | 0 | 0 | 2 | 0 | 0 | 3 | 0 | 4 | 25 | 34 |
| Jinghong | 6 | 16 | 19 | 22 | 20 | 34 | 19 | 16 | 10 | 18 | 22 | 21 | 15 | 8 | 15 | 44 | 50 | 355 |
| Menghai | 0 | 5 | 69 | 164 | 75 | 74 | 30 | 33 | 71 | 59 | 94 | 61 | 34 | 29 | 57 | 125 | 151 | 1131 |
| Mengla | 0 | 0 | 3 | 15 | 12 | 20 | 9 | 24 | 33 | 46 | 51 | 44 | 45 | 32 | 33 | 89 | 72 | 528 |
| Dali | 0 | 0 | 2 | 2 | 0 | 8 | 12 | 18 | 45 | 19 | 76 | 52 | 50 | 56 | 84 | 82 | 68 | 574 |
| Yangbi | 0 | 1 | 0 | 0 | 0 | 0 | 2 | 0 | 0 | 0 | 2 | 6 | 0 | 1 | 2 | 4 | 2 | 20 |
| xiangyun | 110 | 50 | 17 | 20 | 2 | 3 | 3 | 0 | 1 | 1 | 1 | 1 | 3 | 1 | 1 | 2 | 0 | 216 |
| Binchuan | 0 | 1 | 0 | 0 | 0 | 0 | 0 | 3 | 0 | 2 | 1 | 1 | 6 | 2 | 0 | 2 | 5 | 23 |
| Midu | 1 | 1 | 6 | 13 | 20 | 11 | 11 | 32 | 28 | 1 | 3 | 1 | 17 | 17 | 7 | 13 | 8 | 190 |
| Nanjian | 0 | 0 | 0 | 0 | 0 | 2 | 0 | 2 | 0 | 0 | 2 | 3 | 2 | 1 | 0 | 2 | 3 | 17 |
| Weishan | 0 | 0 | 0 | 1 | 0 | 0 | 0 | 0 | 0 | 0 | 2 | 0 | 0 | 0 | 3 | 2 | 8 | 16 |
| Yongping | 0 | 0 | 0 | 0 | 0 | 0 | 1 | 0 | 1 | 0 | 1 | 0 | 1 | 1 | 2 | 1 | 1 | 9 |
| Yunlong | 0 | 0 | 0 | 0 | 1 | 2 | 1 | 1 | 3 | 1 | 9 | 8 | 5 | 7 | 6 | 7 | 9 | 60 |
| Eryuan | 0 | 7 | 6 | 1 | 2 | 3 | 1 | 0 | 1 | 2 | 3 | 3 | 5 | 4 | 5 | 3 | 3 | 49 |
| Jianchuan | 0 | 0 | 0 | 0 | 0 | 0 | 0 | 0 | 0 | 0 | 0 | 0 | 0 | 0 | 1 | 3 | 2 | 6 |
| Heqing | 2 | 0 | 0 | 0 | 0 | 0 | 1 | 1 | 0 | 0 | 1 | 1 | 0 | 1 | 0 | 0 | 2 | 9 |
| Mang | 0 | 0 | 0 | 1 | 11 | 66 | 52 | 41 | 68 | 189 | 259 | 297 | 363 | 328 | 407 | 689 | 650 | 3421 |
| Ruili | 0 | 0 | 0 | 0 | 2 | 6 | 2 | 10 | 3 | 6 | 10 | 9 | 17 | 46 | 34 | 51 | 42 | 238 |
| Longchuan | 2 | 0 | 0 | 0 | 1 | 13 | 13 | 31 | 128 | 111 | 194 | 222 | 228 | 218 | 308 | 464 | 463 | 2396 |
| Lianghe | 0 | 0 | 2 | 0 | 0 | 2 | 12 | 17 | 70 | 74 | 127 | 149 | 126 | 91 | 100 | 122 | 146 | 1038 |
| Yingjiang | 0 | 1 | 0 | 48 | 103 | 137 | 115 | 160 | 206 | 180 | 240 | 262 | 320 | 284 | 341 | 383 | 428 | 3208 |
| Lushui | 0 | 0 | 0 | 0 | 0 | 1 | 3 | 3 | 5 | 3 | 3 | 2 | 6 | 6 | 11 | 6 | 20 | 69 |
| Fugong | 0 | 0 | 0 | 0 | 1 | 1 | 1 | 0 | 1 | 5 | 16 | 32 | 51 | 39 | 67 | 103 | 141 | 458 |
| Lanping | 0 | 0 | 0 | 0 | 1 | 0 | 0 | 0 | 0 | 9 | 18 | 15 | 58 | 39 | 40 | 60 | 66 | 306 |
| Gongshan | 1 | 1 | 0 | 0 | 0 | 0 | 0 | 1 | 0 | 1 | 4 | 4 | 13 | 4 | 4 | 6 | 17 | 56 |
| Xiangge | 0 | 0 | 0 | 0 | 0 | 0 | 0 | 0 | 0 | 0 | 0 | 0 | 1 | 1 | 0 | 1 | 1 | 4 |
| Weixi | 0 | 0 | 0 | 0 | 0 | 1 | 0 | 0 | 0 | 0 | 1 | 0 | 0 | 0 | 7 | 7 | 9 | 25 |
| Deqin | 0 | 0 | 0 | 0 | 0 | 0 | 0 | 0 | 0 | 0 | 0 | 0 | 1 | 0 | 0 | 0 | 1 | 2 |
| Total | 290 | 347 | 499 | 1001 | 1117 | 1289 | 1798 | 2562 | 3690 | 3052 | 4979 | 6144 | 8598 | 6564 | 7907 | 9995 | 11236 | 71068 |

Tabel 4.Average population of Yunnan Province (10,000 person), 2006-2022

| Prefecture | 2006 | 2007 | 2008 | 2009 | 2010 | 2011 | 2012 | 2013 | 2014 | 2015 | 2016 | 2017 | 2018 | 2019 | 2020 | 2021 | 2022 |
| --- | --- | --- | --- | --- | --- | --- | --- | --- | --- | --- | --- | --- | --- | --- | --- | --- | --- |
| Kunming | 612 | 617 | 622 | 626 | 636 | 646 | 651 | 656 | 660 | 665 | 670 | 676 | 682 | 690 | 771 | 848 | 860 |
| Qujing | 568 | 571 | 576 | 580 | 584 | 588 | 592 | 596 | 599 | 603 | 607 | 610 | 614 | 617 | 597 | 573 | 576 |
| Yuxi | 223 | 226 | 227 | 228 | 230 | 231 | 232 | 234 | 235 | 236 | 237 | 238 | 238 | 239 | 232 | 225 | 228 |
| Baoshan | 245 | 245 | 246 | 247 | 249 | 252 | 253 | 255 | 256 | 257 | 259 | 261 | 262 | 263 | 253 | 242 | 240 |
| Zhaotong | 510 | 519 | 527 | 532 | 528 | 524 | 528 | 532 | 536 | 541 | 545 | 551 | 556 | 562 | 537 | 505 | 495 |
| Lijiang | 121 | 121 | 122 | 122 | 124 | 125 | 126 | 127 | 127 | 128 | 128 | 129 | 129 | 130 | 128 | 125 | 124 |
| Puer | 257 | 257 | 258 | 258 | 257 | 255 | 257 | 258 | 259 | 260 | 261 | 262 | 263 | 264 | 253 | 239 | 237 |
| Lincang | 236 | 237 | 238 | 239 | 241 | 244 | 246 | 247 | 249 | 250 | 251 | 252 | 253 | 254 | 240 | 225 | 224 |
| Chuxiong | 266 | 268 | 269 | 270 | 269 | 270 | 271 | 272 | 273 | 273 | 274 | 274 | 275 | 275 | 259 | 240 | 237 |
| Honghe | 433 | 436 | 439 | 443 | 447 | 452 | 455 | 458 | 461 | 464 | 467 | 470 | 473 | 476 | 463 | 446 | 441 |
| Wenshan | 338 | 340 | 342 | 344 | 349 | 353 | 355 | 357 | 359 | 360 | 361 | 363 | 365 | 366 | 359 | 347 | 345 |
| Banna | 105 | 106 | 107 | 107 | 111 | 114 | 115 | 115 | 115 | 116 | 117 | 118 | 118 | 119 | 125 | 131 | 131 |
| Dali | 348 | 348 | 349 | 350 | 348 | 347 | 349 | 350 | 352 | 354 | 355 | 357 | 359 | 361 | 348 | 333 | 331 |
| Dehong | 116 | 117 | 118 | 119 | 120 | 122 | 122 | 124 | 125 | 127 | 129 | 130 | 131 | 132 | 132 | 132 | 132 |
| Nujiang | 52 | 53 | 53 | 53 | 54 | 54 | 54 | 54 | 54 | 54 | 54 | 55 | 55 | 56 | 56 | 55 | 55 |
| Diqing | 37 | 37 | 38 | 38 | 39 | 40 | 40 | 41 | 41 | 41 | 41 | 41 | 41 | 41 | 39 | 39 | 39 |
| Total | 4467 | 4499 | 4529 | 4557 | 4586 | 4616 | 4645 | 4673 | 4700 | 4728 | 4756 | 4786 | 4815 | 4844 | 4790 | 4706 | 4693 |

Tabel 5. Average population of Yunnan Province (10,000 person), 2006-2022

| Counties | 2006 | 2007 | 2008 | 2009 | 2010 | 2011 | 2012 | 2013 | 2014 | 2015 | 2016 | 2017 | 2018 | 2019 | 2020 | 2021 | 2022 |
| --- | --- | --- | --- | --- | --- | --- | --- | --- | --- | --- | --- | --- | --- | --- | --- | --- | --- |
| Anning | 31 | 32 | 32 | 32 | 33 | 34 | 35 | 35 | 36 | 36 | 37 | 38 | 38 | 39 | 44 | 48 | 49 |
| Binchuan | 34 | 34 | 34 | 34 | 35 | 35 | 35 | 35 | 35 | 36 | 36 | 36 | 36 | 37 | 36 | 34 | 34 |
| Cangyuan | 17 | 17 | 17 | 17 | 18 | 18 | 18 | 18 | 19 | 19 | 19 | 19 | 19 | 19 | 18 | 16 | 16 |
| Changning | 34 | 34 | 34 | 34 | 34 | 35 | 35 | 35 | 35 | 35 | 35 | 36 | 36 | 36 | 34 | 32 | 31 |
| Chenggong | 22 | 22 | 23 | 23 | 27 | 31 | 32 | 32 | 33 | 33 | 33 | 34 | 35 | 36 | 51 | 66 | 69 |
| Chengjiang | 16 | 16 | 16 | 16 | 17 | 17 | 17 | 17 | 17 | 18 | 18 | 18 | 18 | 18 | 18 | 17 | 18 |
| Chuxiong | 54 | 55 | 55 | 55 | 57 | 59 | 59 | 59 | 60 | 60 | 60 | 60 | 60 | 60 | 62 | 63 | 62 |
| Daguan | 25 | 25 | 26 | 26 | 26 | 26 | 27 | 27 | 27 | 27 | 28 | 28 | 28 | 28 | 25 | 21 | 20 |
| Dali | 62 | 62 | 63 | 64 | 65 | 65 | 66 | 66 | 66 | 67 | 67 | 67 | 68 | 68 | 73 | 77 | 77 |
| Dayao | 29 | 29 | 29 | 29 | 28 | 27 | 28 | 28 | 28 | 28 | 28 | 28 | 28 | 28 | 26 | 23 | 22 |
| Deqin | 6 | 6 | 6 | 6 | 6 | 7 | 7 | 7 | 7 | 7 | 7 | 7 | 7 | 7 | 6 | 5 | 6 |
| Dongchuan | 29 | 29 | 29 | 29 | 28 | 27 | 27 | 28 | 28 | 28 | 28 | 28 | 28 | 28 | 27 | 26 | 26 |
| Eryuan | 16 | 16 | 16 | 16 | 16 | 16 | 16 | 16 | 17 | 17 | 17 | 17 | 17 | 17 | 16 | 14 | 15 |
| Eshan | 28 | 28 | 28 | 28 | 27 | 27 | 27 | 27 | 27 | 27 | 28 | 28 | 28 | 28 | 27 | 25 | 24 |
| Fengqing | 45 | 45 | 45 | 46 | 46 | 46 | 46 | 47 | 47 | 47 | 47 | 47 | 47 | 48 | 43 | 38 | 38 |
| Fugong | 9 | 9 | 10 | 10 | 10 | 10 | 10 | 10 | 10 | 10 | 10 | 10 | 10 | 10 | 11 | 11 | 11 |
| Fuming | 15 | 15 | 15 | 15 | 15 | 15 | 15 | 15 | 15 | 15 | 15 | 16 | 16 | 16 | 15 | 15 | 15 |
| Funing | 39 | 39 | 40 | 40 | 40 | 41 | 41 | 41 | 42 | 42 | 42 | 42 | 42 | 42 | 41 | 39 | 39 |
| Fuyuan | 70 | 70 | 70 | 70 | 71 | 73 | 73 | 73 | 74 | 74 | 74 | 74 | 75 | 75 | 71 | 67 | 67 |
| Gejiu | 45 | 46 | 46 | 46 | 46 | 46 | 46 | 47 | 47 | 47 | 47 | 47 | 47 | 46 | 44 | 42 | 42 |
| Gengma | 23 | 28 | 28 | 28 | 29 | 30 | 30 | 30 | 30 | 31 | 31 | 31 | 31 | 31 | 30 | 28 | 28 |
| Gongshan | 4 | 4 | 4 | 4 | 4 | 4 | 4 | 4 | 4 | 4 | 4 | 4 | 4 | 4 | 4 | 4 | 4 |
| Guandu | 17 | 17 | 17 | 17 | 19 | 21 | 21 | 21 | 22 | 22 | 22 | 22 | 22 | 22 | 26 | 29 | 27 |
| Guangnan | 71 | 75 | 75 | 76 | 81 | 86 | 86 | 87 | 87 | 88 | 88 | 89 | 91 | 93 | 127 | 161 | 162 |
| Gucheng | 76 | 76 | 77 | 77 | 78 | 79 | 79 | 80 | 80 | 80 | 81 | 81 | 81 | 82 | 79 | 76 | 76 |
| Hekou | 10 | 10 | 10 | 10 | 10 | 11 | 11 | 11 | 11 | 11 | 11 | 11 | 11 | 11 | 11 | 10 | 10 |
| Heqing | 26 | 27 | 27 | 27 | 26 | 26 | 26 | 26 | 26 | 26 | 26 | 26 | 27 | 27 | 26 | 24 | 24 |
| Honghe | 28 | 29 | 29 | 29 | 29 | 30 | 30 | 30 | 30 | 31 | 31 | 31 | 31 | 31 | 30 | 28 | 27 |
| Hongta | 46 | 47 | 47 | 48 | 49 | 50 | 50 | 50 | 50 | 51 | 51 | 51 | 51 | 52 | 55 | 59 | 60 |
| Huaning | 21 | 21 | 21 | 21 | 22 | 22 | 22 | 22 | 22 | 22 | 22 | 22 | 22 | 22 | 21 | 19 | 19 |
| Huaping | 16 | 16 | 16 | 16 | 17 | 17 | 17 | 17 | 17 | 17 | 17 | 17 | 18 | 18 | 17 | 16 | 16 |
| Huize | 88 | 88 | 89 | 91 | 91 | 91 | 92 | 92 | 93 | 93 | 94 | 94 | 95 | 95 | 86 | 79 | 79 |
| Jianchuan | 52 | 52 | 52 | 53 | 53 | 53 | 54 | 54 | 54 | 55 | 55 | 55 | 55 | 56 | 54 | 53 | 53 |
| Jiangcheng | 18 | 18 | 18 | 18 | 17 | 17 | 17 | 17 | 17 | 17 | 18 | 18 | 18 | 18 | 17 | 16 | 16 |
| Jiangchuan | 12 | 12 | 12 | 12 | 12 | 12 | 12 | 12 | 13 | 13 | 13 | 13 | 13 | 13 | 12 | 11 | 11 |
| Jianshui | 27 | 28 | 28 | 28 | 28 | 28 | 28 | 28 | 28 | 29 | 29 | 29 | 29 | 29 | 27 | 25 | 26 |
| Jingdong | 33 | 34 | 34 | 35 | 35 | 36 | 36 | 36 | 37 | 37 | 37 | 37 | 38 | 37 | 35 | 33 | 32 |
| Jinggu | 28 | 28 | 28 | 28 | 28 | 28 | 29 | 29 | 30 | 30 | 30 | 30 | 31 | 31 | 33 | 35 | 35 |
| Jinghong | 38 | 38 | 38 | 38 | 37 | 36 | 36 | 36 | 36 | 37 | 37 | 37 | 37 | 37 | 34 | 30 | 30 |
| Jinning | 31 | 31 | 31 | 31 | 30 | 29 | 29 | 29 | 30 | 30 | 30 | 30 | 30 | 30 | 29 | 28 | 27 |
| Jinping | 47 | 48 | 48 | 48 | 50 | 52 | 53 | 53 | 53 | 53 | 54 | 54 | 54 | 55 | 60 | 64 | 65 |
| Kaiyuan | 31 | 31 | 31 | 31 | 32 | 32 | 33 | 33 | 33 | 33 | 33 | 34 | 34 | 33 | 33 | 32 | 32 |
| Lancang | 21 | 21 | 21 | 21 | 21 | 21 | 21 | 22 | 22 | 22 | 22 | 22 | 22 | 22 | 21 | 20 | 19 |
| Lanping | 50 | 50 | 50 | 50 | 50 | 49 | 50 | 50 | 50 | 50 | 50 | 50 | 50 | 51 | 48 | 44 | 44 |
| Lianghe | 16 | 16 | 16 | 16 | 16 | 15 | 16 | 16 | 16 | 16 | 16 | 16 | 16 | 16 | 15 | 14 | 14 |
| Linxiang | 30 | 30 | 30 | 31 | 32 | 32 | 33 | 33 | 33 | 33 | 34 | 34 | 34 | 34 | 36 | 37 | 37 |
| Longchuan | 27 | 27 | 27 | 28 | 28 | 28 | 28 | 28 | 28 | 29 | 29 | 29 | 29 | 29 | 28 | 27 | 27 |
| Longling | 88 | 88 | 89 | 89 | 92 | 94 | 95 | 95 | 96 | 96 | 97 | 97 | 98 | 98 | 94 | 90 | 90 |
| Longyang | 18 | 18 | 18 | 18 | 18 | 18 | 18 | 18 | 19 | 19 | 19 | 19 | 20 | 20 | 19 | 18 | 19 |
| Ludian | 18 | 18 | 19 | 19 | 19 | 19 | 19 | 19 | 19 | 19 | 19 | 19 | 19 | 19 | 20 | 20 | 20 |
| Lufeng | 39 | 39 | 39 | 39 | 40 | 40 | 41 | 41 | 41 | 41 | 42 | 42 | 42 | 43 | 41 | 38 | 37 |
| Luliang | 39 | 38 | 38 | 38 | 39 | 39 | 40 | 40 | 40 | 41 | 41 | 42 | 42 | 43 | 41 | 40 | 39 |
| Luoping | 61 | 61 | 62 | 62 | 62 | 62 | 63 | 63 | 63 | 64 | 64 | 64 | 65 | 65 | 62 | 60 | 60 |
| Luquan | 44 | 44 | 44 | 44 | 43 | 43 | 43 | 43 | 43 | 43 | 43 | 43 | 43 | 43 | 40 | 36 | 36 |
| Lushui | 43 | 44 | 44 | 45 | 42 | 40 | 40 | 40 | 41 | 41 | 41 | 41 | 41 | 41 | 40 | 38 | 38 |
| Luxi | 45 | 54 | 54 | 55 | 55 | 55 | 56 | 56 | 56 | 56 | 57 | 57 | 58 | 59 | 57 | 53 | 53 |
| Lvchun | 37 | 21 | 22 | 22 | 22 | 22 | 22 | 23 | 23 | 23 | 23 | 23 | 24 | 24 | 23 | 21 | 20 |
| Maguan | 24 | 27 | 28 | 28 | 28 | 28 | 28 | 28 | 28 | 28 | 29 | 29 | 29 | 29 | 27 | 24 | 24 |
| Malipo | 32 | 36 | 36 | 37 | 37 | 37 | 37 | 37 | 37 | 38 | 38 | 38 | 38 | 38 | 35 | 31 | 31 |
| Malong | 27 | 19 | 19 | 19 | 19 | 19 | 19 | 19 | 19 | 19 | 19 | 19 | 20 | 20 | 20 | 19 | 19 |
| Mang | 28 | 37 | 38 | 38 | 39 | 39 | 39 | 40 | 40 | 41 | 41 | 42 | 42 | 43 | 43 | 45 | 46 |
| Menghai | 39 | 40 | 40 | 40 | 41 | 42 | 42 | 42 | 43 | 43 | 44 | 45 | 46 | 48 | 55 | 59 | 59 |
| Mengla | 33 | 33 | 33 | 33 | 33 | 33 | 34 | 34 | 34 | 34 | 34 | 34 | 35 | 35 | 35 | 35 | 36 |
| Menglian | 25 | 26 | 26 | 26 | 27 | 28 | 28 | 29 | 29 | 29 | 29 | 29 | 29 | 30 | 30 | 31 | 31 |
| Mengzi | 13 | 13 | 13 | 13 | 13 | 14 | 14 | 14 | 14 | 14 | 14 | 14 | 14 | 14 | 14 | 14 | 14 |
| Midu | 32 | 32 | 32 | 32 | 32 | 31 | 32 | 32 | 32 | 32 | 32 | 32 | 32 | 32 | 29 | 26 | 26 |
| Mile | 52 | 52 | 53 | 53 | 54 | 54 | 55 | 55 | 55 | 56 | 56 | 56 | 57 | 57 | 55 | 54 | 54 |
| Mojiang | 38 | 38 | 38 | 38 | 37 | 36 | 36 | 36 | 37 | 37 | 37 | 37 | 37 | 37 | 33 | 28 | 28 |
| mouding | 20 | 20 | 21 | 21 | 21 | 21 | 21 | 21 | 21 | 21 | 21 | 21 | 21 | 21 | 18 | 15 | 15 |
| Nanhua | 24 | 24 | 24 | 24 | 24 | 24 | 24 | 24 | 24 | 24 | 24 | 24 | 24 | 24 | 22 | 20 | 20 |
| Nanjian | 22 | 22 | 22 | 22 | 22 | 21 | 21 | 21 | 22 | 22 | 22 | 22 | 22 | 22 | 21 | 19 | 19 |
| Ninger | 19 | 19 | 19 | 20 | 19 | 19 | 19 | 19 | 19 | 19 | 19 | 19 | 19 | 19 | 18 | 16 | 16 |
| Ninglang | 25 | 25 | 26 | 26 | 26 | 26 | 26 | 26 | 26 | 27 | 27 | 27 | 27 | 27 | 26 | 24 | 24 |
| Panlong | 64 | 65 | 66 | 69 | 77 | 81 | 82 | 82 | 82 | 83 | 83 | 84 | 84 | 84 | 92 | 99 | 101 |
| Pingbian | 15 | 15 | 15 | 15 | 15 | 15 | 15 | 16 | 16 | 16 | 15 | 15 | 15 | 16 | 14 | 13 | 12 |
| Qiaojia | 68 | 69 | 69 | 70 | 72 | 75 | 75 | 76 | 76 | 76 | 77 | 77 | 78 | 78 | 89 | 100 | 101 |
| Qilin | 51 | 53 | 54 | 53 | 52 | 52 | 52 | 52 | 53 | 53 | 54 | 54 | 54 | 55 | 51 | 46 | 45 |
| Qiubei | 46 | 46 | 46 | 46 | 47 | 48 | 48 | 48 | 49 | 49 | 49 | 49 | 49 | 50 | 48 | 46 | 46 |
| Ruili | 16 | 17 | 17 | 17 | 18 | 18 | 19 | 19 | 19 | 20 | 20 | 21 | 21 | 21 | 24 | 25 | 23 |
| Shidian | 37 | 38 | 38 | 38 | 39 | 39 | 40 | 40 | 40 | 40 | 41 | 41 | 41 | 41 | 39 | 37 | 38 |
| Shilin | 32 | 32 | 32 | 33 | 32 | 31 | 31 | 31 | 31 | 31 | 32 | 32 | 32 | 32 | 31 | 29 | 28 |
| Shiping | 24 | 24 | 24 | 24 | 25 | 25 | 25 | 25 | 26 | 26 | 26 | 26 | 26 | 27 | 25 | 24 | 24 |
| Shizong | 29 | 29 | 29 | 30 | 30 | 30 | 30 | 30 | 31 | 31 | 31 | 31 | 31 | 32 | 29 | 27 | 26 |
| Shuangbai | 16 | 16 | 16 | 16 | 16 | 16 | 16 | 16 | 16 | 16 | 16 | 16 | 16 | 16 | 15 | 13 | 13 |
| shuangjiang | 23 | 18 | 18 | 18 | 18 | 18 | 18 | 18 | 18 | 18 | 18 | 18 | 19 | 19 | 18 | 16 | 16 |
| Shuifu | 9 | 10 | 10 | 10 | 10 | 10 | 10 | 10 | 10 | 11 | 11 | 11 | 11 | 11 | 11 | 10 | 10 |
| Simao | 26 | 26 | 26 | 26 | 28 | 30 | 30 | 31 | 31 | 31 | 31 | 32 | 32 | 32 | 37 | 42 | 42 |
| Songming | 34 | 35 | 35 | 32 | 29 | 29 | 29 | 29 | 30 | 31 | 31 | 32 | 34 | 35 | 38 | 41 | 41 |
| suijiang | 15 | 16 | 16 | 16 | 16 | 15 | 16 | 16 | 16 | 16 | 16 | 16 | 16 | 16 | 15 | 13 | 13 |
| Tengchong | 63 | 63 | 63 | 64 | 64 | 65 | 65 | 66 | 66 | 66 | 67 | 67 | 67 | 67 | 66 | 64 | 64 |
| Tonghai | 30 | 30 | 30 | 30 | 30 | 30 | 31 | 31 | 31 | 31 | 31 | 31 | 31 | 31 | 30 | 29 | 29 |
| Weishan | 37 | 37 | 38 | 38 | 38 | 39 | 39 | 39 | 40 | 40 | 40 | 41 | 41 | 41 | 38 | 35 | 34 |
| Weixi | 31 | 31 | 31 | 31 | 31 | 31 | 31 | 31 | 31 | 31 | 31 | 31 | 32 | 31 | 29 | 27 | 26 |
| Weixin | 15 | 15 | 15 | 15 | 16 | 16 | 16 | 16 | 16 | 16 | 16 | 16 | 17 | 16 | 15 | 15 | 15 |
| Wenshan | 44 | 44 | 45 | 45 | 47 | 48 | 49 | 49 | 49 | 50 | 50 | 50 | 50 | 51 | 57 | 62 | 63 |
| Wuding | 88 | 88 | 88 | 87 | 86 | 86 | 86 | 86 | 86 | 87 | 87 | 87 | 88 | 88 | 101 | 115 | 117 |
| Wuhua | 27 | 28 | 28 | 28 | 28 | 27 | 28 | 28 | 28 | 28 | 28 | 28 | 28 | 28 | 26 | 24 | 23 |
| Xiangge | 25 | 25 | 25 | 25 | 25 | 26 | 26 | 26 | 26 | 26 | 26 | 26 | 26 | 27 | 24 | 20 | 19 |
| xiangyun | 9 | 9 | 9 | 9 | 9 | 9 | 9 | 9 | 9 | 9 | 9 | 10 | 10 | 10 | 9 | 9 | 9 |
| Xichou | 72 | 69 | 70 | 71 | 73 | 76 | 77 | 77 | 77 | 78 | 78 | 79 | 79 | 80 | 88 | 96 | 98 |
| Ximeng | 16 | 16 | 16 | 16 | 17 | 17 | 18 | 18 | 18 | 18 | 18 | 18 | 18 | 18 | 18 | 19 | 19 |
| Xinping | 46 | 45 | 45 | 46 | 46 | 46 | 46 | 46 | 46 | 47 | 47 | 47 | 47 | 47 | 44 | 41 | 40 |
| Xishan | 28 | 28 | 28 | 28 | 29 | 29 | 29 | 29 | 29 | 29 | 29 | 29 | 29 | 29 | 28 | 26 | 26 |
| Xuanwei | 132 | 133 | 133 | 134 | 132 | 131 | 131 | 132 | 133 | 134 | 136 | 138 | 138 | 138 | 129 | 118 | 119 |
| Xundian | 50 | 50 | 51 | 51 | 49 | 46 | 46 | 46 | 47 | 47 | 47 | 47 | 47 | 48 | 47 | 46 | 46 |
| Yangbi | 37 | 37 | 37 | 38 | 37 | 37 | 37 | 38 | 38 | 38 | 39 | 39 | 39 | 39 | 36 | 32 | 31 |
| Yanjin | 45 | 45 | 45 | 46 | 46 | 47 | 47 | 47 | 47 | 47 | 48 | 48 | 48 | 48 | 48 | 47 | 48 |
| Yanshan | 11 | 11 | 10 | 10 | 10 | 10 | 10 | 10 | 10 | 10 | 11 | 11 | 11 | 11 | 10 | 10 | 10 |
| Yaoan | 21 | 21 | 21 | 21 | 20 | 20 | 20 | 20 | 20 | 20 | 20 | 20 | 21 | 21 | 19 | 16 | 16 |
| Yiliang | 41 | 42 | 42 | 43 | 42 | 42 | 42 | 43 | 43 | 43 | 44 | 44 | 44 | 44 | 42 | 38 | 38 |
| Yiliang | 53 | 54 | 54 | 55 | 54 | 52 | 53 | 53 | 54 | 54 | 55 | 55 | 56 | 57 | 54 | 50 | 49 |
| Yimen | 18 | 18 | 18 | 18 | 18 | 18 | 18 | 18 | 18 | 18 | 18 | 18 | 18 | 18 | 17 | 15 | 15 |
| Yingjiang | 29 | 29 | 30 | 30 | 30 | 31 | 31 | 31 | 31 | 32 | 32 | 32 | 32 | 33 | 31 | 30 | 30 |
| Yongde | 36 | 36 | 37 | 37 | 37 | 37 | 37 | 38 | 38 | 38 | 38 | 38 | 38 | 38 | 36 | 33 | 33 |
| Yongping | 18 | 18 | 18 | 18 | 18 | 18 | 18 | 18 | 18 | 18 | 18 | 18 | 18 | 18 | 17 | 16 | 16 |
| Yongren | 11 | 11 | 11 | 11 | 11 | 11 | 11 | 11 | 11 | 11 | 11 | 11 | 11 | 11 | 11 | 10 | 10 |
| Yongshan | 40 | 40 | 39 | 40 | 40 | 40 | 40 | 40 | 41 | 41 | 41 | 42 | 42 | 42 | 39 | 35 | 34 |
| Yongsheng | 39 | 40 | 40 | 40 | 40 | 39 | 40 | 40 | 40 | 40 | 40 | 40 | 41 | 41 | 37 | 34 | 34 |
| Yuanjiang | 23 | 23 | 23 | 23 | 22 | 22 | 22 | 22 | 22 | 22 | 22 | 22 | 22 | 22 | 22 | 22 | 22 |
| Yuanmou | 21 | 21 | 22 | 22 | 22 | 22 | 22 | 22 | 22 | 22 | 22 | 22 | 22 | 22 | 21 | 19 | 20 |
| Yuanyang | 21 | 21 | 21 | 21 | 22 | 22 | 22 | 22 | 22 | 22 | 22 | 22 | 22 | 22 | 21 | 20 | 20 |
| Yulong | 38 | 38 | 39 | 39 | 40 | 40 | 40 | 40 | 41 | 41 | 41 | 42 | 42 | 42 | 39 | 35 | 35 |
| Yun | 44 | 44 | 44 | 45 | 45 | 45 | 45 | 46 | 46 | 46 | 46 | 46 | 47 | 47 | 43 | 39 | 39 |
| Yunlong | 21 | 21 | 21 | 21 | 20 | 20 | 20 | 20 | 20 | 21 | 21 | 21 | 21 | 21 | 20 | 18 | 18 |
| Zhanyi | 40 | 40 | 40 | 41 | 42 | 43 | 44 | 44 | 44 | 45 | 45 | 45 | 45 | 46 | 43 | 40 | 40 |
| Zhaoyang | 75 | 77 | 80 | 80 | 80 | 79 | 80 | 81 | 81 | 82 | 83 | 84 | 85 | 86 | 89 | 90 | 89 |
| zhenkang | 17 | 17 | 17 | 17 | 17 | 18 | 18 | 18 | 18 | 18 | 18 | 18 | 19 | 19 | 18 | 17 | 17 |
| Zhenxiong | 127 | 131 | 136 | 138 | 136 | 133 | 134 | 135 | 137 | 138 | 139 | 140 | 142 | 143 | 139 | 134 | 131 |
| Zhenyuan | 21 | 21 | 21 | 21 | 21 | 21 | 21 | 21 | 21 | 21 | 21 | 21 | 21 | 22 | 20 | 18 | 18 |
| Total | 4467 | 4499 | 4529 | 4557 | 4586 | 4617 | 4645 | 4673 | 4701 | 4728 | 4756 | 4786 | 4815 | 4844 | 4790 | 4706 | 4693 |
