# Supplementary material for: Retrospective analysis of spatiotemporal variation of scrub typhus in Yunnan Province, 2006–2022
Source: PLoS Negl Trop Dis. 2024 Dec 10;18(12):e0012654. doi: 10.1371/journal.pntd.0012654 (PMC11630589; doi:10.1371/journal.pntd.0012654)
Supplement: Attachment — Submitted filename: Reviewer report_Plos NTD.pdf [file pntd.0012654.s010.pdf]

Reviewer report
Title: Retrospective analysis of spatiotemporal variation of scrub typhus in Yunnan Province, 2006-2022

Title: OK
Abstract
Line 10: The word 'saw' is not the correct term to use in this context.
Line 11: Suggest avoiding starting a sentence with a number in formal writing.
Line 12: ….are concentrated in June to November...
Line 23-24: This sentence is repeated from the above. Please check again.

Introduction
Overall, the introduction was good.
Line 79-84: This sentence is too long with many 'and'. Just provide the objectives of this study.
Line 80: Please correct the spelling.

Materials and methods.
The methodology part was very good, straightforward and easy to understand.
Line 153-157: This sentence is too long. It would be better to separate it into a few shorter sentences for clarity.
Line 189: Remove the comma.

Results
Results presented very well with tables, figures, and maps to understand.
Line 217: Suggest avoiding starting a sentence with a number in formal writing.

Discussion:
Overall, the discussion was ok. However, some of the discussion was very general and did not reflect the current situation in the counties of Yunnan Province.
Line 278-283: The statements here do not align with the data collected during the COVID-19 season. The data in the S6 file did not show a reduction in scrub typhus cases; instead, it continues to increase.
Line 285-289: This sentence is too long. Please rephrase.
Line 292-294: All these factors are generally described. Please relate them to the counties most affected, particularly those in the results, such as the southern and southwestern regions.
Line 304-306: This sentence doesn't sound right. Please improve to 'The geographic variation in the distribution of chigger mites, which carry scrub typhus, may contribute to the observed differences in seasonal patterns.'
Line 306-307: There have been no previous sentences related to *Leptotrombidium*. Please write in full and italicise them.
Line 330-333: This sentence doesn't sound correct. Please correct the sentence.
Line 331: Please delete the non-cited reference.
Line 332 and 334: Is *Rattus tanezumi* the only rodent species reported in Yunnan? Please italicize the sentence.
Line 378: Check the spelling
Line 379: Write Scrub typhus in a small letter
Line 391: There is another conclusion sub-chapter below.
Line 402-404: Typically, adults are actively engaged in agricultural activities. Please improve the sentence
Line 407: What do you mean by scrub typhus season?

Line 412-415: Please combine this into one shorter and clearer sentence.

Conclusion:
The conclusion was acceptable; however, it was separated from the last paragraph of the discussion. Please clarify the conclusion.
