# Supplementary material for: Retrospective analysis of spatiotemporal variation of scrub typhus in Yunnan Province, 2006–2022
Source: PLoS Negl Trop Dis. 2024 Dec 10;18(12):e0012654. doi: 10.1371/journal.pntd.0012654 (PMC11630589; doi:10.1371/journal.pntd.0012654)
Supplement: Attachment — Submitted filename: Response letter.doc [file pntd.0012654.s007.doc]

**Response letter**

Thank you very much for your time and consideration about our article entitled “Retrospective analysis of spatiotemporal variation of scrub typhus in Yunnan Province, 2006-2022” (ID: PNTD-D-23-01544). We are grateful for the constructive and insightful comments from you and the reviewers to help us improve our work. We have enclosed point-to-point responses to reviewers’ comments in the online submitting system and revised the manuscript accordingly. Based on the reviewers' suggestions, we revised the manuscript. We corrected spelling errors in the manuscript, revised limitations, added a correlation analysis between scrub typhus and meteorological factors in the Results section, and revised the Methods and Discussion sections accordingly. In order to increase the clarity of the figures, we have re-uploaded the figures and updated S3 and S4 Appendix. We have made adjustments to the authorship order based on the contributions' magnitude in accordance with the revised manuscript. The changes to the main manuscript text are marked in red and blue in the tracked-change version uploaded as a supplementary file.

Thank you for considering our manuscript for publication on your esteemed journal, and we appreciate the opportunity to revise and improve our work based on the feedback received.

**Response to Reviewer 1’s comments**

**Methods**

**=============**

**The study describes a spatio temporal analysis using surveillance data from the Yunnan province in China. The study design, the population described, sample size and statistical analysis seem adequate to consider the manuscript for publication.**

**Response:**Thank you for your recognition of our research and methods.

**Results**

**=============**

**The results presented are as per analysis plan and the presentation of images are of sufficient clarity.**

**Response:** Thank you for your affirmation of our research results.

**Conclusions**

**=============**

**The limitations described and conclusions are adequate and the public health importance of the study has been adequately addressed.**

**Response:** Thank you for your confirmation of our discussion and conclusions of the research.

**Editorial and Data Presentation Modifications?**

**=============**

**There are a few** **spelling errors such as the word 'shrub typhus' used instead of 'scrub typhus'.**

**Response:** Thank you for your comments. We apologize for any errors in spelling due to our oversight, which may have affected your reading experience. We have rechecked the spelling throughout the article and corrected the mistakes (please see Lines 328).

**Summary and General Comments**

**=============**

**Overall, the study uses secondary data to analyse the spatio-temporal distribution of scrub typhus in the Yunnan province in China over a period of 16 years. The study is well conducted and presented.**

**Response:** Thank you for your affirmation of our research. We have made corrections to the spelling errors in the article, which further enhances the quality of our manuscript. Once again, thank you for taking the time to review it.

**Response to Reviewer 2’s comments**

**Methods**

**=============**

**Although often used for diagnosis, the Weil-Felix test has poor specificity. Please include a statement of this as a limitation of the data. It would also be helpful to include the proportion of results from this method and how this taken into account in defining cases.**

**Response:** Thank you for your valuable suggestions! We recognize that the specificity of the Weil-Felix test is insufficient, which may result in a certain rate of false positives in the test outcomes. We have stated this in the limitations (please see Lines 356-361). Moreover, since our data is obtained from surveillance data from the Center for Disease Control and Prevention (CDC), we cannot obtain the proportion of patients diagnosed by the Weil-Felix test.

The case definition was based on the unified diagnostic criteria formulated by the Chinese Ministry of Health. Diagnosis is made based on epidemiological history, clinical manifestations, and laboratory results. Our study excluded suspected cases. 69,799 clinically diagnosed cases and 1269 laboratory-confirmed cases were included in our study. The specific diagnostic criteria are as follows (Detailed diagnostic criteria are provided in S2.).

1.1 Epidemiological History

During the peak season, individuals who have been in or traveled to scrub typhus endemic areas within the preceding 3 weeks and have a history of outdoor activities, mainly involving fieldwork, rural fishing, camping, lying or sitting on grass, and contact with straw, should be considered for scrub typhus.

1.2 Clinical Manifestations

1.2.1 Fever

1.2.2 Lymph node enlargement

1.2.3 Rash

1.2.4 Specific eschar or ulcer

1.3 Laboratory Investigations

1.3.1 Positive Weil-Felix test: Single serum with OXK titer ≥1:160;

1.3.2 Positive Indirect Immunofluorescence Assay: Four-fold or higher rise in IgG antibody titer in paired sera;

1.3.3 Positive PCR (Polymerase Chain Reaction) for scrub typhus DNA;

1.3.4 Isolation of the causative pathogen.

2.1 Suspected Cases

Individuals meeting criteria 1.1 and 1.2.1, plus either 1.2.2 or 1.2.3, with the exclusion of other diseases; or individuals without a clear epidemiological history but presenting with all three criteria 1.2.1, 1.2.2, and 1.2.3 during the peak season.

2.2 Clinical Diagnosis Cases

Suspected cases plus criterion 1.2.4; or individuals meeting all three criteria 1.1, 1.2.1, and 1.2.4.

2.3 Laboratory Diagnosis Cases

Suspected cases plus any of the criteria 1.3.2, 1.3.3, or 1.3.4; or clinical diagnosis cases plus any of the criteria 1.3.1, 1.3.2, 1.3.3, or 1.3.4.

**Revision in the manuscript:**

Lines 356-361: Our study has some limitations because the data is derived from the surveillance data of the Yunnan Provincial Center for Disease Control and Prevention, which does not reveal the laboratory methods used in case diagnosis. The current use of different laboratory methods exhibits inconsistent specificity and sensitivity, particularly the specificity and sensitivit of the Weil Felix test are not high enough. This could result in some false positive and false negative rates.

**Results**

**=============**

**Results are presented well.**

**Response:** Thank you for your recognition of our research results.

**Conclusions**

**=============**

**Please include the proportion of the positive results received by Weil Felix test, and address the limitation of the use of this test due to its low specificity and how this was accounted for in the analysis.**

**Response:** Thank you for your suggestion. I'm sorry, due to the fact that our data is sourced from surveillance data from the CDC, we are unable to obtain the proportion of the positive results received by Weil Felix test. We stated this in the limitation (please see Lines 356-364).

Our case definition is based on epidemiological history, clinical manifestations, and laboratory results, and suspected cases were excluded. Furthermore, while reading the literature, we found some studies showing that the Weil Felix test has low sensitivity, but it is not low specificity, especially when the titer ≥1:80. Therefore, there is reason to believe that our data is reliable overall. Some of the literature is as follows.

1.	Kannan K, John R, Kundu D, et al: Performance of molecular and serologic tests for the diagnosis of scrub typhus. PLoS Negl Trop Dis 2020, 14(11):e0008747.

2.	Ritu GP, Arif W, Sihag KK, et al: Comparative Evaluation of Different Tissues and Molecular Techniques for the Zoonotic Surveillance of Scrub Typhus. Vector Borne Zoonotic Dis 2024.

3.	Vanramliana, Pautu L, Lalmalsawma P, et al: Epidemiology of scrub typhus and other rickettsial infections (2018-22) in the hyper-endemic setting of Mizoram, North-East India. PLoS Negl Trop Dis 2023, 17(11):e0011688.

4.	Banerjee A, Kulkarni S: Orientia tsutsugamushi: The dangerous yet neglected foe from the East. Int J Med Microbiol 2021, 311(1):151467.

**Revision in the manuscript:**

Lines 356-364 Our study has some limitations because the data is derived from the surveillance data of the Yunnan Provincial Center for Disease Control and Prevention, which does not reveal the laboratory methods used in case diagnosis. The current use of different laboratory methods exhibits inconsistent specificity and sensitivity, particularly the specificity and sensitivit of the Weil Felix test are not high enough. This could result in some false positive and false negative rates. In addition, scrub typhus is classified as "other" infectious diseases and is not included in the list of legal infectious diseases in mainland China, leading to underreporting issues. These may lead to discrepancies between our data and the real situation.

**Editorial and Data Presentation Modifications?**

**=============**

**none**

**Response:** Thank you for your recognition of our manuscript.

**Summary and General Comments**

**=============**

**The authors do a good job of describing the incidence of scrub typhus in the Yunnan Province, from 2006-2022 with relevant discussion of the analysis. The lab methods in use are standard testing methods of varying specificities and the data could be strengthened by including these as a limitation (specifically regarding the Weil Felix test) or acknowledging how this was taken int account for case definition and analysis.**

**Response:** Thank you for recognizing our research and for your suggestions, your suggestions are very helpful to our research. The laboratory methods used in case definition are inconsistent, with varying specificities, especially the Weil Felix test may lead to a certain degree of false positives due to its insufficient specificity. We have declared this in the limitations (please see Lines 356-364).

**Revision in the manuscript:**

Lines 356-364: Our study has some limitations because the data is derived from the surveillance data of the Yunnan Provincial Center for Disease Control and Prevention, which does not reveal the laboratory methods used in case diagnosis. The current use of different laboratory methods exhibits inconsistent specificity and sensitivity, particularly the specificity and sensitivit of the Weil Felix test are not high enough. This could result in some false positive and false negative rates. In addition, scrub typhus is classified as "other" infectious diseases and is not included in the list of legal infectious diseases in mainland China, leading to underreporting issues. These may lead to discrepancies between our data and the real situation.

**Response to Reviewer 3’s comments**

**Summary and General Comments**

**=============**

**1.This paper presents a relatively standard analysis of scrub typhus case data from Yunnan Province of China. As is, I cannot tell what we've learned from this analysis. Some simple statistical approaches are applied to case data across administrative units and over time. There is an argument for some novelty in the measurement of the "****migration of the center of gravity of scrub typhus". However, it isn't clear why this is an important thing to do. It also appears to be a geostatistical approach that is meant for point data that is here being applied to geometric centroids of administrative units. While this latter critique may be minor, it should be addressed, and I stress that it isn't clear why it is important to look at the movement of the 'center of gravity'. Case data are already being reported throughout this region - it seems best to keep doing that rather than try to focus more case detection based on this statistic that might be spuriously moving because of the shapes of the administrative units.**

**Response:** Thank you for your comments and suggestions, which have greatly assisted us in further enhancing the quality of our manuscript. We conducted a spatiotemporal analysis of scrub typhus in Yunnan Province from 2006 to 2022. It provides an understanding of the overall trends in scrub typhus, including the number of cases, high prevalence areas and transmission dynamics, and high prevalence seasons, which can help to identify areas and seasons that require increased surveillance and intervention (Avoid risky behaviors during peak seasons and in high-risk areas). This helps governments and health organizations formulate targeted prevention and control strategies and allocate relevant resources.

The importance of studying the migration of the center of gravity of scrub typhus is that it can reveal dynamic patterns of scrub typhus transmission. It provides a dynamic view of how diseases change spatially over time and allows for the timely identification of new trends in disease transmission. Although the shape of the administrative unit may affect the location of the center of mass, the analysis was conducted using the incidence of scrub typhus in each location as a weight to reduce this error. Our objective is to uncover the transmission dynamics of scrub typhus, rather than merely focusing on the areas where the center of gravity is located. The center of gravity of the incidence of scrub typhus in Yunnan Province has shifted to the south and west, indicating that the incidence of the disease is higher in the southern and western parts of Yunnan Province, thus leading to a shift in the center of gravity. While attention should be paid to scrub typhus in Yunnan Province, more attention should be paid to the south and west of the province.

Center of gravity migration trajectory analysis is used in many research fields. In economics research, it can be used to study the change of industrial structure, the trend of economic growth, the direction of urban development, etc., providing reference for the formulation of macroeconomic policies. In sociological research, it can analyze the changing patterns of social phenomena such as population migration and urbanization processes. In epidemiology, it can analyze transmission pathways and trends.

The following is the literature on the application of this method in brucellosis:

1.	Liang P, Zhao Y, Zhao J, et al: The spatiotemporal distribution of human brucellosis in mainland China from 2007-2016. BMC Infect Dis 2020, 20(1):249.

2.	Zhang M, Chen X, Bu Q, et al: Spatiotemporal dynamics and influencing factors of human brucellosis in Mainland China from 2005-2021. BMC Infect Dis 2024, 24(1):76.

**2.Also, it is curious that scrub typhus is not a reported disease in this setting, but that the authors come to a conclusion that changes in reporting cannot be driving the patterns that are evident in space and time.**

**Response:** We apologize for any misunderstanding due to the lack of precision in our writing. On January 1, 2004, the China CDC established a network-based, real-time direct reporting system for infectious diseases, the China National Notifiable Infectious Disease Reporting Information System(CNNDS). Scrub typhus was included in it since 2006. The use of surveillance systems may lead to an increase in reports of scrub typhus during a certain period. But the change in the incidence rate of scrub typhus in Yunnan Province significantly exceeded the national level. From 2006 to 2022, the incidence of scrub typhus in Yunnan Province increased from 0.65 per 100,000 population to 23.94 per 100,000 population. However, the incidence rate of scrub typhus in mainland China increased from 0.09 per 100,000 population to 1.60 per 100,000 population between 2006 and 2016. In addition, between 2006 and 2013, the overall incidence of reportable infectious diseases in China remained relatively stable. It is reasonable to assume that the impact of monitoring changes is to some extent equal across localities. Therefore, changes in surveillance may not be the main reason for the rise in scrub typhus incidence in Yunnan Province. Relying solely on reported changes cannot fully explain the significant temporal and spatial variations of scrub typhus in Yunnan Province. Therefore, we also discussed other potential factors that might have contributed to the increase in the incidence and geographical expansion of scrub typhus in later discussions.

**Revision in the manuscript:**

Lines 259-268: Scrub typhus has been monitored through the CNNDS, a web-based, real-time, direct reporting system for infectious diseases since 2006. The use of passive surveillance systems and the increased use of diagnostic tools may lead to an increase in scrub typhus reporting rates. But between 2006 and 2013, the overall incidence of reportable infectious diseases in China remained relatively stable[14]. In addition, the change in the incidence rate of scrub typhus in Yunnan Province significantly exceeded the national level. Therefore, changes in surveillance may not be the main reason for the rise in scrub typhus incidence in Yunnan Province. Relying solely on reported changes cannot fully explain the significant temporal and spatial variations of scrub typhus in Yunnan Province.

**3.Finally, this paper would seriously benefit from further analyses and contextualization. It seems that cases are occurring more frequently in the 'summer'. Does this correspond to the rainy season? How about an analysis that looks at rainfall patterns, or surface water (NDWI, etc.), and reported case incidence? How about looking at other environmental factors? Descriptive epidemiology is important, especially for under-studied diseases, but this paper currently reads as though it was a report being written for local health authorities.**

**Response:** Thank you for your valuable suggestions, which will help us to improve the quality of our manuscripts! We performed a Spearman analysis of the relationship between scrub typhus cases and mean temperature, precipitation, and relative humidity. Significant positive correlations between the number of scrub typhus cases and average temperature, precipitation and relative humidity. High temperatures and rainfall in summer and fall in Yunnan Province may have contributed to the pattern of summer and fall scrub typhus in Yunnan Province. In addition, in the discussion section, we added a look at future research. Although this manuscript is of public health significance for factors that do not further the study of scrub typhus, it is still of public health significance. The analysis provides up-to-date spatial and temporal epidemiological characteristics of scrub typhus in Yunnan Province, which can provide recommendations for targeted surveillance, prevention, and health education. It may also provide clues for further research on influencing factors and transmission dynamics of scrub typhus. I think it's an integral part.

**Revision in the manuscript****:**

(1) Lines 244-249: **Results:** The results showed that the peaks in the number of scrub typhus cases were generally consistent with the peaks in average temperature, precipitation, and relative humidity. Correlation analysis showed significant positive correlations between the number of scrub typhus cases and average temperature, precipitation and relative humidity. Spearman correlation coefficients (rs) were 0.522, 0.585, 0.761, respectively (Figure 7).
(2) Lines 294-298: **Discussion:** Our analysis shows that scrub typhus cases in Yunnan Province are associated with higher temperatures, relative humidity, and precipitation. Yunnan has abundant rainfall, high humidity and temperature in summer and fall, which is suitable for the growth of chiggers. This may also contribute to the seasonality of scrub typhus.
(3) Lines 284-289: **Discussion:** A Study has revealed positive correlations between temperature, relative humidity, precipitation, and scrub typhus incidence. Another study shows that temperature, humidity, and rainfall have a nonlinear impact and a lag effect on the prevalence of scrub typhus in southwest Yunnan. High temperatures, high humidity, and heavy rainfall increase the risk of scrub typhus.
(4) Lines 394-397: **Discussion:** Follow-up studies should further explore the reasons influencing the increase in the incidence of scrub typhus and the changes in spatial and temporal distribution. Influence of host, environmental and socio-economic factors on scrub typhus, transmission dynamics of scrub typhus, etc.
