# Supplementary material for: Retrospective analysis of spatiotemporal variation of scrub typhus in Yunnan Province, 2006–2022
Source: PLoS Negl Trop Dis. 2024 Dec 10;18(12):e0012654. doi: 10.1371/journal.pntd.0012654 (PMC11630589; doi:10.1371/journal.pntd.0012654)
Supplement: Attachment — Submitted filename: respond letter.doc [file pntd.0012654.s008.doc]

**Response letter**

Thank you very much for your time and consideration about our article entitled “Retrospective analysis of spatiotemporal variation of scrub typhus in Yunnan Province, 2006-2022” (ID: PNTD-D-23-01544). We are grateful for the constructive and insightful comments from you and the reviewers to help us improve our work. We have enclosed point-to-point responses to reviewers’ comments in the online submitting system and revised the manuscript accordingly. Based on the reviewers' suggestions, we revised the manuscript. The changes to the main manuscript text are marked in red and blue in the tracked-change version uploaded as a supplementary file.

Thank you for considering our manuscript for publication on your esteemed journal, and we appreciate the opportunity to revise and improve our work based on the feedback received.

**Response to Reviewer 2’s comments**

1. In the discussion, authors describe “healthcare workers should be sensitized to this disease and consider the possibility of scrub typhus when unexplained fever occurs in highly endemic areas”. Please consider and address the need for education and awareness efforts at the community level. Are there public health awareness efforts? If so, what are they and please state the possible effect of these efforts.

**Response:** Thank you for your advice. We believe that education at the community level can increase public awareness. Enabling people to understand the symptoms, transmission routes, and preventive methods of scrub typhus helps them to detect potential infections in a timely manner. Secondly, to sensitize healthcare workers to scrub typhus and enable more accurate diagnosis and treatment. At present, there is publicity about scrub typhus relying on media platforms such as the WeChat official account of the CDC, allowing more residents to understand the hazards of this diseases. Learning effective precautions, such as avoiding prolonged time in mite habitats such as grass and woods, wearing long-sleeved clothing and long pants, and using insect repellents, can reduce the risk of infection.

2. Line 106. Describe what is meant by “suspicious cases”

**Response:**Thank you for your suggestion, we have added after " suspected cases " in line 109, (S2 Appendix). The definition of suspected case is written in detail in annex 2, and due to the large number of words, there is no explanation in line 109.

Suspected Cases:

Individuals meeting criteria 1.1 and 1.2.1, plus either 1.2.2 or 1.2.3, with the exclusion of other diseases; or individuals without a clear epidemiological history but presenting with all three criteria 1.2.1, 1.2.2, and 1.2.3 during the peak season.

1.1During the peak season, individuals who have been in or traveled to scrub typhus endemic areas within the preceding 3 weeks and have a history of outdoor activities, mainly involving fieldwork, rural fishing, camping, lying or sitting on grass, and contact with straw, should be considered for scrub typhus.

1.2.1 Fever

1.2.2 Lymph node enlargement

1.2.3 Rash

1.2.4 Specific eschar or ulcer

3. Line 106-108. Were travel related cases considered/counted as where they may have become infected or only where the patient held residence?

**Response:** Thank you for your question. We categorize cases according to the address where the case resides in Yunnan Province, which is the current address.

4. Line 109-110. Please state the proportion of cases that were diagnosed clinically, and laboratory confirmed.

**Response:** Thanks to your suggestion, we have added the number of cases with clinically diagnosed and laboratory-confirmed cases (please see Line 113-114).

**Revision in the manuscript:**

Line 113-114: 69,799 clinically diagnosed cases and 1269 laboratory-confirmed cases.

5. Line 28. 29. Please use Italics with O. tsutsugamushi

**Response:** Thank you. We have italicized *O. tsutsugamushi* in the manuscript (please see Line 28.29).

6. Line 44. Replace “triggered” with “caused by”

**Response:** Thank you, we have made changes in the manuscript according to your comments (please see Line 45).

7. Lines 48-50. Describe/state the percentage of patients have eschars?

**Response:** Thanks to your suggestion. We have added the percentage of patients have eschars (please see Line 51).

**Revision in the manuscript:**

Line 51: The percentage of patients with eschar ranges from 7% to 97%.

8. Line 62-63. Describe what is considered “rapid and timely diagnostic methods” and how is it different from existing methods like PCR?

**Response:** Thank you for your question and we apologize for any misunderstanding you may have had due to our expression. What we mean is that the specificity and sensitivity of the Weil-Felix test is not high enough, and IFA, PCR is not performed in all hospitals. Lack of simple, rapid, reliable and cost-effective diagnostic methods. We have rewritten this sentence in the manuscript (please see Line 64-65).

**Revision in the manuscript:**

Lines 64-65: There is a lack of simple, rapid, reliable and cost-effective diagnostic methods and effective protective vaccines.

9. Line 65-67. Check redundancy in sentence.

**Response:** Thanks to your suggestions. We have revised the sentences in the manuscript (please see Line 66-69).

**Revision in the manuscript:**

Line 66-69: Especially in the last two decades, the geographic distribution of Scrub typhus in mainland China has expanded dramatically. The incidence rate has risen dramatically from 0.09 per 100,000 population in 2006 to 1.60 per 100,000 population in 2016

10. Line 72. Why do you expect to see an increase in Yunnan Providence as indicated?

**Response:** Thank you for your question. We came up with this statement because the reference suggests that given the trends identified in their study, findings suggest that scrub typhus incidence in Yunnan providence is likely to continue to increase in the future. We apologize for any misunderstanding you may have had due to our inaccurate presentation, which we have corrected in the manuscript (please see Line 73-74).

**Revision in the manuscript:**

Line 73-74: Study suggests that the number of cases in Yunnan Province may continue to increase in the future.

**Response to Reviewer 4’s comments**

1. The abstract lacks a conclusion part and needs to include one during the submission process.

**Response:**Thank you for your review. The abstract in our manuscript included a conclusion part that may have been unclear due to formatting, which we have adjusted.

2. Line 95: "Data collection" should be adjusted as a sub-title.

**Response:** Thanks to your suggestion, we have changed "Data collection" to a sub-title (please see Line 98).

3. Line 109: "Province" should be written as "province."

**Response:** Thanks to your suggestion, we've changed "Province" to "province" in the manuscript (please see Line 111).

4. Line 113: The link provided does not work.

**Response:** Thanks to your suggestion, we have replaced the new link in the manuscript (please see Line 116).

5. Line 115: Change "from" to "for."

**Response:** Thank you, we have made changes in the manuscript according to your comments (please see Line 118).

6. Figure S1: Suggest rewording the legend name “states.”

**Response:** Thank you, we have made changes the legend name of Figure S1 according to your comments.

7. Line 122: Suggest making it clearer how the 2022 population was calculated.

**Response:** Thanks to your suggestion, we have updated the description of the population calculation for 2022 in the manuscript (please see Line 125-127).

**Revision in the manuscript:**

Line 125-127: Since the population at the end of 2022 has not been announced, the average population in 2022 is replaced by the year-end population in 2021.

8. Line 138: If there is no specific method or reason for these classes, please remove this sentence as readers can understand it through the map.

**Response:** Thanks to your suggestion, we’ve removed that sentence from the manuscript.

9. Line 141: Reword to "Then, using the Spatial Trend Analysis module, disease trends were plotted on a three-dimensional spatial map to reflect the overall changes in cases in both the latitudinal and longitudinal directions of the regional unit."

**Response:** Thank you very much for your corrections to the poorly expressed sentences in our manuscript, which we have corrected in the manuscript (please see Line 142-144).

10. Figure 1: Suggest capitalizing the legend name “Incidence rate.”

**Response:** Thank you, we have changed the name of the legend for Figure 1 to "Incidence rate".

11. Figure 5: Legend name not capitalized.

**Response:** Thank you, we have made changes the legend name of Figure 5.

12. Results, Meteorological factors analysis: It seems the authors only conducted a Spearman correlation test between average temperature, precipitation, relative humidity, and scrub typhus cases. Suggest using more advanced methods to explore their associations on a finer spatial scale, like the county level. Additionally, consider expanding the analysis to include geographic and socio-economic factors. The regional heterogeneity could be driven and explained by multiple factors, which would be interesting to see.

**Response:**Thank you very much, this is great advice. We conducted Spearman correlation tests only between mean temperature, precipitation, relative humidity, and scrub typhus case. Due to the limitations of our data, we were unable to obtain information on other geographical and socio-economic factors, as well as more detailed meteorological data at a finer spatial scale. Therefore, we did not conduct an analysis at a finer spatial scale and only performed correlations. In the future, if more detailed and diverse data can be obtained, we will be very glad to conduct further in-depth and detailed research to explore the heterogeneity affecting the incidence of scrub typhus in Yunnan Province and the factors causing the increase in the incidence rate. At the same time, we hope that our research can provide a few clues for the analysis of the influencing factors of scrub typhus incidence and the dynamics of scrub typhus transmission in Yunnan Province.

13. Since Yunnan province shares borders with Myanmar, Laos, and Vietnam, it would be valuable to include results from imported cases.

**Response:**Thank you for your valuable suggestions. In our study, we included foreign nationals living in Yunnan Province, that is, cases whose current address is in Yunnan. Because cases whose current address is not in Yunnan cannot be analyzed when conducting spatio-temporal analysis, we excluded cases whose current residential address is not in Yunnan.

14. Discussion, lines 259 to 268: Although the authors mentioned that the overall incidence of reported infectious diseases in China remained relatively stable, this cannot lead to the conclusion that changes in reporting do not explain the variation of scrub typhus in Yunnan. The overall stable incidence of infectious diseases reported nationwide does not necessarily mean that the reporting rate of scrub typhus in a specific region (Yunnan) is also stable. Suggest reconsidering this paragraph.

**Response:** Thank you for your suggestion. After thinking about it, we think your proposal is correct. We have made the revisions and deletions in the manuscript (please see Line 260-264).

**Revision in the manuscript:**

Line 260-264: Scrub typhus has been monitored through the CNNDS, a web-based, real-time, direct reporting system for infectious diseases since 2006. The use of passive surveillance systems and the increased use of diagnostic tools may lead to an increase in scrub typhus reporting rates. This could be one reason for the increase in scrub typhus incidence in Yunnan Province[15].

15. Line 306: Lack of reference.

**Response:** Thank you for the reminder, we have inserted references.

16. Line 305: "study" should be lowercase, not "Study."

**Response:** Thank you for your careful review, we have changed "Study" to "study" (please see Line 306).

17. Discussion, lines 299 to 323: These lines all refer to other studies without describing this study’s association analysis. Suggest adding more analysis on how the county-level case numbers relate to local meteorological and other factors to enrich the results and support the discussion.

**Response:**Thank you for your advice. We have added to the discussion a description of the results of our correlation analysis. We apologize that due to data limitations, we were unable to add more analysis to illustrate the relationship between the number of cases at the county level and local climate and other factors. The reason for writing about the other studies is to discuss the possible reasons affecting the increase in the number of cases as much as possible, in order to provide a little bit of ideas for future related studies in Yunnan Province (please see Line 306-312).

**Revision in the manuscript:**

Line 306-312: A study has revealed positive correlations between temperature, relative humidity, precipitation, and scrub typhus incidence[41]. This is consistent with our results. Our results showed that the number of scrub typhus cases was significantly and positively correlated with average temperature, precipitation and relative humidity. Another study shows that temperature, humidity, and rainfall have a nonlinear impact and a lag effect on the prevalence of scrub typhus in southwest Yunnan[42]. High temperatures, high humidity, and heavy rainfall increase the risk of scrub typhus[42].

18.Discussion, lines 358-361: Need reference.

**Response:** Thanks to your reminder, we have inserted references in the manuscript.

19.Lines 361-363: To my knowledge, in 2006, scrub typhus was added as a reporting disease in the National Notifiable Infectious Disease Reporting Information System at the Chinese Centers for Disease Control and Prevention (China CDC). Please clarify this.

**Response:** Thanks to your suggestion, we have added "Scrub typhus has been included in the CNNDS since 2006." to the manuscript. As this point has already been addressed in lines 269-272 of our discussion, we will simply add here, "Scrub typhus has been included in the CNNDS since 2006." (please see Line 364-367).

**Revision in the manuscript:**

Lines 364-367: In addition, Scrub typhus has been included in CNNDS since 2006. But scrub typhus is classified as "other" infectious diseases and is not included in the list of legal infectious diseases in mainland China, leading to underreporting issues.

20.Line 369: "In general, the study's findings can represent the real situation of this disease in Yunnan Province." Suggest deleting this. It is not proper to say this after a whole paragraph of limitations.

**Response:** Thanks to your suggestion, we have removed this sentence from the manuscript.

22.Since the study period covered the COVID-19 period, suggest adding a statement about whether there was any impact of COVID-19.

**Response:** Thanks for the suggestion, it's very helpful for our article! We added this part to the discussion (please see Line 264-269).

**Revision in the manuscript:**

Lines 264-269: In addition, during the COVID-19 pandemic, there was a similarity between the clinical manifestations of scrub typhus and certain COVID-19 symptoms, which may have led to a decline in the diagnosis and detection rate of scrub typhus[29]. At the same time, the closure measures and restrictions on outdoor activities implemented in response to the pandemic may have had an objective effect in reducing the incidence of scrub typhus[30].

23.Why is the title of S6 "date"? Should it be "data"?

**Response:** Thank you for your careful review, and we apologize that due to our carelessness we wrote the "data" as "date", and we have corrected the name of S6 and the corresponding place in the manuscript.
